# Developing and implementing an obesity medicine fellowship program: Experience at a U.S. academic medical center

**DOI:** 10.1016/j.obpill.2025.100204

**Published:** 2025-08-27

**Authors:** Zoobia W. Chaudhry, Marci Laudenslager, Kimberly A. Gudzune, Selvi Rajagopal

**Affiliations:** a Johns Hopkins University School of Medicine, Baltimore, MD, USA; b Johns Hopkins Bloomberg School of Public Health, Baltimore, MD, USA

**Keywords:** Obesity management, Curriculum, Graduate medical education

## Abstract

**Background:**

Obesity is a prevalent and complex chronic disease yet physicians’ training in obesity care is often inadequate. Fellowship training in obesity medicine is now available at multiple academic medical centers; however, there has been limited description and evaluation of these programs to date. Therefore, this article describes our curriculum development process and fellowship program implementation experience at a U.S. academic medical center.

**Methods:**

This was a structured, multi-phase curriculum development study guided by established obesity medicine competencies and certification standards. We used a six-step approach to curriculum development to guide our design with content informed by the Obesity Medicine Education Collaborative competencies, American Board of Obesity Medicine (ABOM) content outline, and Accreditation Council for Graduate Medical Education - International Obesity Medicine Fellowship program requirements. We utilized several tools of instruction as well as modes of assessing learners. Feedback from fellows and faculty was used to iteratively refine the program over the initial 3 years.

**Results:**

We identified 19 content domains key for obesity medicine. Within each domain, we created instructional components including didactic e-lectures, directed readings, reflective assignments, role play activities, and clinical experiences. We developed evaluation tools to determine trainee progress including knowledge check assessments, mini-clinical evaluation exercises (mini-CEXs), and structured feedback. Early outcomes indicate that trainees achieve competence in obesity care, engage in meaningful scholarly activity, and benefit from professional development opportunities. All graduating fellows have successfully achieved ABOM certification. Feedback has informed fellowship refinements, particularly improvements in rotation timing and duration as well as additional subspecialty elective options.

**Conclusion:**

This obesity medicine fellowship curriculum provides a structured, multidisciplinary program and graduates have achieved competency in obesity care. Obesity medicine fellowship training holds promise in advancing the field through innovative education and leadership development.

## Graphical abstract

**Figure:**
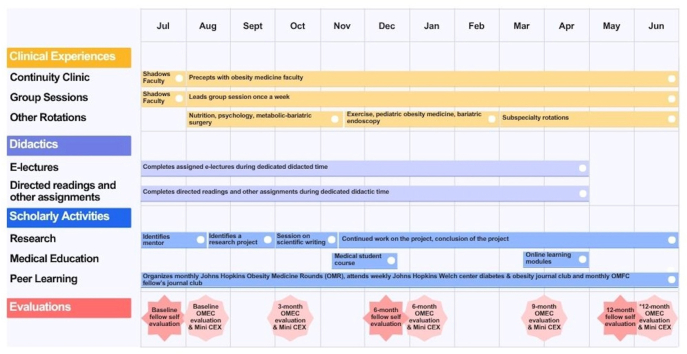

## 1 Introduction

The prevalence of obesity among U.S. adults has steadily increased since the 1980s [1,2]. Despite broad recognition of obesity as a complex, chronic disease, physician training in its management remains limited. A recent systematic review found a global lack of structured obesity education across all levels of medical training, though implemented programs consistently improve learner outcomes [3]. This gap has clinical consequences: physicians report low self-efficacy, initiate fewer evidence-based treatments [4], and may perpetuate weight bias in care [5].

In general, fellowship training advances clinical expertise and may support scholarship and leadership among physicians – physicians’ pursuit of fellowship training has increased steadily [6]. Fellowship-trained physicians also deliver higher quality care, adhere to guidelines, and achieve better results than physicians without this training [7,8]. For example, research among cardiologists has found that implantable cardioverter-defibrillator procedures performed by cardiologists who were not fellowship-trained in electrophysiology were associated with a higher risk of procedural complications than when the devices were implanted by an electrophysiologist [9]. Similar to other fields of medicine, physicians may pursue fellowship training in obesity medicine, which is a pathway to certification in obesity medicine offered by the American Board of Obesity Medicine (ABOM) [10]. The Obesity Medicine Fellowship Council (OMFC) was established in 2018 [11]. While OMFC currently supports over 25 obesity medicine fellowship programs, the organization does not offer accreditation. There has been limited description and evaluation of these obesity medicine fellowship programs to date.

This article describes the development of a comprehensive, competency-based obesity medicine fellowship curriculum, its integration into a postgraduate training program, and methods for evaluating its implementation and impact. It aims to serve as a practical guide for program directors and educators seeking to strengthen training in obesity medicine.

## 2 Overview of curriculum development process

We developed an obesity medicine fellowship curriculum following strategies from *Curriculum Development for Medical Education: A Six-Step Approach* [12], which includes: (1) problem identification and general needs assessment; (2) targeted needs assessment; (3) goals and objectives; (4) educational strategies; (5) implementation; and (6) evaluation and feedback. In the following sections, we describe our process for each step. All faculty involved in the curriculum development completed a curriculum development course focused on this approach as well as principles from Kolb's model of experiential learning [13]. These individuals formed an education committee to develop, maintain and enhance the fellowship curriculum.

### 2.1 Step 1: Problem identification and general needs assessment

Obesity remains one of the most prevalent and undertreated chronic diseases in the United States [1,2,14]. However, there is limited availability of rigorous education in obesity care during medical school, residency, and fellowship training for physicians [3]. This lack of education has been hypothesized to contribute to physicians’ low self-efficacy in treating obesity and reduces their likelihood of initiating evidence-based treatment strategies [4]. Although the need for improved physician training in obesity has been recognized, there remains a lack of competency-based postgraduate curricula needed for clinical mastery.

Fellowship-level training has the potential to address this gap by allowing for focused education in obesity pathophysiology, diagnosis, behavioral interventions, pharmacotherapy, and procedural management as well as and longitudinal clinical experiences where trainees can develop and apply skills in this field. Fellowship training may offer protected time for scholarly activity, interdisciplinary collaboration, and leadership development – essential components for advancing the field of obesity medicine [15]. We identified that a structured fellowship curriculum would be an ideal training model to equip physicians with the comprehensive knowledge and skills required for an obesity medicine physician. As part of this step, we conducted a literature search in PubMed and MedEdPORTAL in 2020 to identify prior descriptions of obesity medicine fellowships; however, we identified no publications – which further highlighted the need to develop a structured, competency-based curriculum aligned with the evolving demands of clinical obesity care and consistent with educational best practices.

### 2.2 Step 2: Targeted needs assessment

Typically, a targeted needs assessment requires engagement with current learners to identify specific educational gaps. Because this was a new program, we did not have existing fellows at the time of initial development. Therefore, we informally engaged trainees rotating through the obesity medicine clinical practice from residency programs in internal medicine, internal medicine-pediatrics and preventive medicine to discuss their knowledge gaps, skills needs, and training preferences. We also considered the existing learning environment and institutional strengths to inform this step. Our institution offers significant resources, including ABOM-certified faculty practicing obesity medicine, clinical programs in metabolic-bariatric surgery and bariatric endoscopy, and an obesity behavioral medicine program. Overall, these sources provided insights into the educational priorities and gaps that informed our targeted needs assessment.

We also leveraged key resources including the ABOM content outline that defines the body of knowledge for the obesity medicine field [[15], [16], [17]], advanced specialty program requirements for graduate medical education in obesity medicine from the Accreditation Council for Graduate Medical Education - International Obesity (ACGME-I) [18], and competencies from the Obesity Medicine Education Collaborative (OMEC) [19,20], to structure the initial curriculum framework. We identified 19 content domains key for obesity medicine. Table 1 identifies these 19 fellowship domains as well as the content from these resources mapped to each domain – the domains include foundational knowledge (e.g., pathophysiology), comprehensive and evidence-based treatment strategies, as well as population health, public health and professionalism. Of note, the initial mapping was informed by the 2015 ABOM examination blueprint [16] and the current curriculum was updated to reflect the 2025 ABOM content outline [17]. The education committee used this framework to guide the development of the fellowship curriculum and fellowship program experiences.

**Table 1:** Mapping obesity medicine educational resources to identify fellowship domains.

2019 OMEC	2024 ACGME International	2025 ABOM	Fellowship Domain
Demonstrate competence in: • Using appropriate language in verbal, nonverbal & written communication that is non-biased, non-judgmental, respectful & empathetic when communicating with patients with obesity (ICS-1) & about patients with obesity with colleagues within one's profession & other members of the healthcare team (ICS-2) • Displaying compassion & respect toward all patients & families who are living with overweight or obesity (P-2) • Advocating for policies that are respectful & free of weight bias (SBP-2)	Demonstrate competence in: • Using appropriate language in verbal, non-verbal & written communication that is non- stigmatizing, non-judgmental, respectful & empathetic when communicating with patients with obesity (ICS-IV.A.1.e).(1).(a)) & about patients with obesity with colleagues within one's profession & other members of the health care team (ICS– IV.A.1.e).(1).(b)) • Displaying compassion & respect toward all patients who have pre-obesity or obesity as well as their families (P-IV.A.1.a).(1).(b)) • Advocating for policies that are respectful & free of weight bias (SBP-IV.A.1.f).(1).(b))	C.3 Reduce Weight Bias & Stigma	**Weight Bias & Stigma**
Demonstrate knowledge of: • Energy homeostasis & weight regulation (MK-2) • Anthropometric measurements & clinical assessments of energy expenditure (MK-3)	Didactics on: • Energy homeostasis & weight regulation across the life course (RSEA-IV.B.3.e) • Anthropometric measurements & clinical assessment of energy expenditure & its application to patient care (RSEA-IV.B.3.a) Demonstrate knowledge of: • Energy homeostasis & weight regulation across the life course (MK-IV.A.1.c).(1).(b)) • Anthropometric assessments & clinical assessments of energy expenditure across the life course (MK-IV.A.1.c).(1).(a))	A.1 Obtain a Weight History	**Weight Regulation & Body Composition**
Demonstrate knowledge of: • Etiologies, mechanisms & biology of obesity (MK-4)	Didactics on: • Etiologies, mechanisms & biology of obesity across the life course (RSEA-IV.B.3.f) Demonstrate knowledge of: • Etiologies, mechanisms & biology of obesity across the life course (MK-IV.A.1.c).(1).(c))	B.1 Discuss Obesity as a Complex Chronic Disease	**Pathophysiology**
Demonstrate knowledge of: • Obesity-related comorbidities & corresponding benefits of body mass index reduction (MK-5)	Didactics on: • Obesity-related comorbidities (RSEA- IV.A.1.(c).(1).e)) Clinical experience in: • Disciplines related to obesity medicine (CE- IV.C.1) Demonstrate knowledge of: • Obesity-related comorbidities & corresponding benefits of weight reduction or weight management (MK- IV.A.1.(c).(1).(e))	A.9 History of Weight-Related Metabolic & Biomechanical Complications A.10 Diagnose Weight-Related Metabolic & Biomechanical Complications B.9 Manage Weight-Related Conditions	**Metabolic & Biomechanical Conditions & Complications**
–	Clinical experience in: • Psychology or mental health (CE- IV.C.6.)	A.7 Neuropsychiatric History A.8 Social History & Social Determinants of Health B.9 Manage Weight-Related Conditions	**Psychological Conditions & Complications**
Demonstrate knowledge of: Etiologies, mechanisms & biology of obesity (MK-4)	Didactics on: • Etiologies, mechanisms, and biology of obesity across the life course including other secondary causes of obesity (RSEA- IV.B.3.f.(2))	A.11 Diagnose Secondary Causes of Weight Gain or Obesity B.8 Tailor Treatment for Special Populations	**Secondary Obesity**
–	Didactics on: • Etiologies, mechanisms & biology of obesity across the life course including genetic determinants (RSEA-IV.B.3.f.(1))	A.12 Diagnose Genetic or Syndromic Obesity B.8 Tailor Treatment for Special Populations	**Genetic & Syndromic Obesity**
Apply knowledge of: • Different cultural views regarding perceptions of desired weight & preferred body shape when communicating with the patient, family & other members of the healthcare team (ICS-3) Demonstrate competence in: • Eliciting comprehensive obesity-focused medical history (PCPS-1) • Performing & documenting comprehensive physical examination for the assessment of obesity (PCPS-2) • Applying clinical reasoning skills when ordering & interpreting appropriate laboratory & diagnostic tests during the evaluation of patients with obesity (PCPS-3)	Apply knowledge of: • Different cultural views regarding perception of desired weight & preferred body shape when communicating with patients, families & other members of the health care team (ICS-IV.A.1.e).(1).(c)) Demonstrate competence in: • Eliciting a comprehensive obesity-focused medical history (PCPS-IV.A.1.b).(1).(a)) • Performing & documenting a comprehensive physical examination for the assessment of obesity (PCPS– IV.A.1.b).(1).(b)) • Applying clinical reasoning skills when ordering & interpreting appropriate laboratory & diagnostic tests during the evaluation of patients who have pre-obesity & obesity (PCPS-IV.A.1.b).(1).(c)) • Use of laboratory evaluation (PCPS– IV.A.1.b).(2).(a)), radiological & other diagnostic procedures (PCPS– IV.A.1.b).(2).(b)), including appropriate test selection for screening, diagnosis & monitoring response to diagnosis & treatment of obesity- related conditions	A.13 Diagnose Obesity & Characterize Its Severity	**Initial Obesity Medicine Evaluation & Assessment**
Apply knowledge of: • Behavioral interventions to develop a comprehensive, personalized, obesity management care plan (MK-10) • Evidence-based models of health behavior change to assess patients' readiness to change to effectively counsel patients for weight management (PCPS-4)	Didactics on: • Behavioral & psychological interventions (RSEA-IV.B.3.b) Clinical experience in: • Psychological management and/or collaborative management with mental health providers (≥2 weeks) (CE-IV.C.6) Apply knowledge of: • Behavioral & psychological interventions in developing a comprehensive, personalized obesity treatment care plan across the life course (MK-IV.A.1.c).(1).(f).(i)) Demonstrate competence in: • Using evidence-based models of health behavior change to assess patients' readiness to change & effectively counsel patients for weight management (PCPS– IV.A.1.b).(1).(d))	A.3 Eating Behavior History A.5 Sleep History B.2 Behavior Modification Counseling	**Behavior Change**
Apply knowledge of: • Nutrition interventions to develop a comprehensive, personalized, obesity management care plan (MK-8)	Didactics on: • Nutrition interventions (RSEA-IV.B.3.g) Clinical experience in: • Nutritional management of patients who have obesity & obesity-related conditions (≥80 h) (CE-IV.C.2) Apply knowledge of: • Nutrition interventions in developing a comprehensive, personalized obesity treatment care plan across the life course (MK-IV.A.1.c).(1).(f).(iii))	A.2 Nutrition History B.3 Nutrition Counseling	**Nutrition**
Apply knowledge of: • Physical activity interventions to develop a comprehensive, personalized, obesity management care plan (MK-9)	Didactics on: • Physical activity interventions (RSEA-IV.B.3.l) Apply knowledge of: • Physical activity interventions in developing a comprehensive, personalized obesity treatment care plan across the life course (MK-IV.A.1.c).(1).(f).(v))	A.4 Physical Activity History B.4 Physical Activity Counseling	**Physical Activity**
Apply knowledge of: • Pharmacological interventions to develop a comprehensive, personalized, obesity management care plan (MK-11)	Didactics on: • Pharmacological management (RSEA-IV.3.k) Apply knowledge of: • Pharmacologic treatments that influence body weight in developing a comprehensive, personalized obesity treatment care plan across the life course (MK-IV.A.1.c).(1).(f).(iv))	A.6 Medication & Supplement History B.5 Obesity Medications	**Pharmacotherapy**
Apply knowledge of: • Emerging treatment modalities to develop a comprehensive, personalized, obesity management care plan (MK-13)	Didactics on: • Emerging obesity treatment modalities (RSEA-IV.3.d) Clinical experience in: • Endoscopic or other minimally invasive bariatric procedures(CE-IV.C.7) Apply knowledge of: • Emerging treatment modalities in developing a comprehensive, personalized obesity treatment care plan across the life course (MK-IV.A.1.c).(1).(f).(ii))	B.6 Bariatric Devices & Non-Surgical Procedures	**Non-Surgical Procedures**
Apply knowledge of: • Surgical treatments of obesity as part of a comprehensive, personalized, obesity management care plan (MK-12)	Didactics on: • Surgical procedures (RSEA- IV.B.3.n) Clinical experience in: • Bariatric & metabolic surgery (≥80 h) (CE-IV.C.3) Apply knowledge of: • Surgical & procedural treatments of obesity in developing a comprehensive, personalized obesity treatment care plan across the life course (MK- IV.A.1.c).(1).(f).(viii))	B.7 Metabolic & Bariatric Surgery	**Surgical Procedures**
Apply knowledge of: • Obesity treatment guidelines to the development of a comprehensive, personalized obesity management care plan (MK-7) Demonstrate competence in: • Engaging patients & their support systems in shared decision-making by incorporating their values & preferences in the development of a comprehensive, personalized obesity management care plan (PCPS-5)	Didactics on: • Obesity treatment guidelines relevant internationally & to the local context (RSEA-IV.B.3.j) Apply knowledge of: • Principles of obesity treatment guidelines in developing a comprehensive, personalized obesity treatment care plan across the life course (MK-IV.A.1.c).(1).(f).(vi)) Demonstrate competence in: • Engaging patients & their support systems in shared decision-making by incorporating their values & preferences in the development of a comprehensive, personalized obesity management care plan (PCPS-IV.A.1.b).(1).(e))	B.8 Tailor Treatment for Special Populations B.10 Formulate an Effective Treatment Plan	**Multicomponent Obesity Medicine Treatment Plan**
–	Clinical experience in: • Providing longitudinal care to a panel of patients throughout their educational program that is supervised by obesity medicine faculty (CE-IV.C.5) • Pediatric obesity medicine (≥80 h) (CE-IV.C.4.	B.11 Manage the Obesity Treatment Plan Long-Term	**Longitudinal Obesity Medicine Care**
Demonstrate competence in: • Analyzing practice systems using quality improvement methods to monitor & optimize obesity care (PBLI-2) • Using information technology related to obesity treatment to optimize delivery of care including electronic health records, software applications & related devices (PBLI-4) • Educating patients, students, residents & other health professionals on the disease of obesity (PBLI-5) • Working collaboratively within an interdisciplinary team dedicated to obesity prevention & treatment strategies (SBP-1)	Demonstrate competence in: • Analyzing practice systems using quality improvement methods to monitor & optimize obesity care (PBLI-IV.A.1.d).(1).(b)) • Using information technology related to obesity treatment to optimize delivery of care, including electronic health records, software applications & related devices (PBLI-IV.A.1.d).(1).(d)) • Educating patients, students, residents & other health professionals about the disease & the assessment, prevention & treatment of obesity (PBLI-IV.A.1.d).(1).(e)) • Working collaboratively within an interdisciplinary team dedicated to obesity prevention & treatment strategies (SBP-IV.A.1.f).(1).(f))	C.2 Manage the Obesity Medicine Practice C.4 Advocate for Patients with Obesity within the Health System	**Health Systems & Population Health**
Demonstrate knowledge in: • Obesity epidemiology (MK-1) • Principles of primary, secondary & tertiary prevention of obesity to the development of a comprehensive obesity management care plan (MK-6) • Costs of obesity intervention & prevention with regards to the individual, healthcare system & community (SBP-4) Demonstrate competence in: • Using chronic disease treatment & prevention models to advance obesity intervention & prevention efforts within the clinical, community & public policy domains (SBP-3)	Didactics on: • Obesity epidemiology • Principles of primary, secondary & tertiary prevention of obesity Demonstrate knowledge of: • Obesity epidemiology (MK-IV.A.1.c).(1).(d)) • Principles of primary, secondary & tertiary prevention of obesity in developing a comprehensive, personalized obesity treatment care plan across the life course (MK-IV.A.1.c).(1).(f).(vii)) • Costs of obesity intervention & prevention with regards to the individual, health care system, & community (SBP-IV.A.1.f).(1).(d)) Demonstrate competence in: • Advocating for health system & public health policies to improve obesity treatment & prevention (SBP-IV.A.1.f).(1).(a)) • Using chronic disease treatment & prevention models to advance obesity intervention & prevention efforts within the clinical, community & public policy domains (SBP-IV.A.1.f).(1).(e))	C.5 Raise Public Awareness of Obesity	**Public Health & Prevention**
Demonstrate competence in: • Evaluating strengths & deficiencies in knowledge of obesity medicine; setting & achieving goals for improvement (PBLI-1) • Using resources to locate, interpret & apply evidence from scientific studies regarding obesity treatment & its comorbidities (PBLI-3) • Demonstrating ethical behavior & integrity when counseling patients & their families who are living with overweight or obesity (P-1)	Didactics on: • Core curriculum in research (SA-IV.D.1.a) Experience in: • Scholarly project under the guidance of a mentor (SA-IV.D.1.b) Demonstrate competence in: • Evaluating strengths & deficiencies in knowledge of obesity medicine; setting & achieving goals for improvement (PBLI-IV.A.1.d).(1).(a)) • Using resources to locate, interpret & apply evidence from scientific studies regarding obesity co-morbidities & treatment (PBLI-IV.A.1.d).(1).(c)) • Critically appraising scientific articles & research methods in the field of obesity medicine (SBP-IV.A.1.f).(1).(c)) • Demonstrating ethical behavior & integrity when counseling patients who have pre-obesity or obesity and their families ((P– IV.A.1.a).(1).(a)))	C.1 Maintain Professional Competence in Obesity Medicine	**Professional Development & Identity as an Obesity Medicine Physician**

Abbreviations: ABOM – American Board of Obesity Medicine; ACGME-I – Accreditation Council for Graduate Medical Education-International; ICS – interpersonal & communications skills; MK – medical knowledge; OMEC – Obesity Medicine Education Collaborative; P – professionalism; PBLI – practice-based learning & improvement; PCPS – patient care & procedural skills; SBP – systems-based practice; RSEA—regularly scheduled educational activities; SA—scholarly activity.

### 2.3 Step 3: Goals and Objectives

For this project, we considered a goal to be a broad statement of the overall fellowship program purpose that included the general capabilities and outcomes it aimed to develop. An objective was a specific, measurable statement of what a fellow should be able to do after instruction. Goals guided curriculum content and priorities, while objectives translated those goals into actionable outcomes, informed instructional methods, and influenced evaluation of both learners and the curriculum.

The goal of the obesity medicine fellowship program is to provide exemplary postgraduate training in evidence-based, multidisciplinary and interprofessional management of obesity and its related diseases and complications. It aims to promote clinical practice innovation, quality improvement, and scholarly work in obesity medicine through close faculty mentorship, diverse research and teaching opportunities for the fellow, and support of each fellow's unique career interests. Building on these goals, we outlined specific curricular objectives to align with the previously identified fellowship domains (Table 2), which are used to guide the fellowship experience. Through this structure, trainees develop the clinical skills and scholarship important for advancing the field of obesity medicine.

**Table 2:** Curricular objectives by fellowship domain.

Fellowship Domain(s)	Associated Curricular Objective(s)
**Weight Bias & Stigma**	• Recognize, address, and mitigate implicit and explicit weight bias within oneself through self-assessment, reflection and communication skills training • Recognize and address implicit and explicit weight bias among staff and other healthcare professionals through education and role modeling respectful and empathetic communication • Recognize and address implicit and explicit weight bias among patients and families through respectful and empathetic communication as well as weight bias internalization through education and support
**Weight Regulation & Body Composition**	• Apply knowledge of weight regulation and body composition to develop individualized care plans for patients with obesity • Integrate advanced diagnostic tools (e.g., bioelectrical impedance analysis, indirect calorimetry) to inform treatment plans
**Pathophysiology**	• Demonstrate knowledge of the etiology, mechanisms, and pathophysiology of obesity across the life course • Integrate diagnostic and staging criteria of obesity into individualized treatment plans
**Metabolic & Biomechanical Conditions & Complications** **Psychological Conditions & Complications**	• Integrate the assessment and clinical management of obesity-related diseases and complications into individualized treatment plans (e.g., hypertension, type 2 diabetes, cardiovascular disease, depression, binge eating disorder)
**Secondary Obesity**	• Demonstrate knowledge of the causes of secondary obesity, including but not limited to endocrine disorders, medications, environmental factors, and social determinants of health • Integrate the assessment and clinical management of secondary obesity into individualized treatment plans
**Genetic & Syndromic Obesity**	• Demonstrate knowledge of etiology, mechanisms, and pathophysiology for genetic and syndromic forms of obesity • Integrate the assessment and clinical management of genetic and syndromic obesity into individualized treatment plans
**Initial Obesity Medicine Evaluation & Assessment**	• Elicit a comprehensive history and perform a thorough medical examination appropriate for an obesity medicine physician • Document a comprehensive history and physical examination relevant for obesity medicine using appropriate clinical standards
**Behavior Change** **Nutrition** **Physical Activity** **Pharmacotherapy** **Non-Surgical Procedures** **Metabolic & Bariatric Surgery**	• Demonstrate proficiency in the assessment of nutrition, physical activity, behavior, sleep and other lifestyle factors relevant to obesity • Apply all evidence-based obesity treatment strategies — including behavior change, nutrition, physical activity, pharmacotherapy, non-surgical procedures, and metabolic-bariatric surgery — to develop comprehensive, individualized care plans • Integrate behavior change, nutrition, physical activity, and pharmacotherapy into treatment plans within shared medical or group visits
**Multicomponent Obesity Medicine Treatment Plan**	• Evaluate obesity treatment guidelines and apply evidence-based lifestyle, pharmacologic, and procedural interventions to create personalized management plans across the life course • Engage effectively with multidisciplinary and interprofessional team members to deliver comprehensive, patient-centered obesity care • Demonstrate proficiency in the management of complex obesity cases, incorporating clinical, psychological, and sociological factors into individualized treatment strategies
**Longitudinal Obesity Medicine Care**	• Demonstrate proficiency in the long-term management of obesity through longitudinal care of a panel of patients
**Health System & Population Health**	• Demonstrate knowledge of how health systems, population health, and policies influence obesity care, access, and outcomes • Engage in academic scholarship through activities such as teaching or quality improvement activities
**Public Health & Prevention**	• Demonstrate understanding of public health strategies for obesity prevention, including community interventions, policy, and social determinants
**Professional Development & Identity as an Obesity Medicine Physician**	• Appraise scientific research and engage in academic scholarship through research activities • Network and identify leadership opportunities through participation in local and national organizations dedicated to obesity or obesity medicine • Achieve certification in obesity medicine from ABOM

Abbreviations: ABOM — American Board of Obesity Medicine.

### 2.4 Step 4: Educational Strategies

Table 3 outlines the curriculum content of our obesity medicine fellowship, employing diverse educational strategies across its core domains to create a comprehensive professional and educational framework for training fellows. For each domain, we developed specific e-lectures, directed readings, clinical experiences, and other educational activities such as workshops, role-play activities, and reflection assignments. To support different learning styles, we elected to use multiple modalities and combined asynchronous learning with faculty-guided discussions.

**Table 3:** Tools of instruction and mode of assessment by fellowship domain.

Fellowship Domain	Tools of Instruction (*Mode of Assessment)*			
	Didactics	Directed Readings	Clinical Experiences	Other Educational Activities^a^
Weight Bias & Stigma	e-Lectures: Selective e-lectures from the Rudd Center for Food Policy & Health. *Weight stigma in healthcare.* University of Connecticut. Retrieved Mar 1st, 2025, from https://supportiveobesitycare.rudd.center.uconn.edu/weight-stigma-in-healthcare/ • What is weight stigma? • Weight stigma in healthcare • How can we improve healthcare for people of all body sizes? • A patient's perspective of weight bias: Insights from Patty Nece (b*Post lecture quiz)*	cChapters from Handbook of Obesity – Volume 1;4th ed: • Puhl RM. Bias, discrimination, and obesity. (pp. 617–636). ^e^Additional Readings: • Rubino F et al. Joint international consensus statement for ending stigma of obesity. Nat Med 26, 485–497 (2020). *(*b*Discussion with Program Director)*	Longitudinal obesity medicine clinic *(OMEC based milestone evaluation and Mini-CEX by obesity medicine faculty)*	Reflection Assignments: The fellow is expected to complete a guided self-reflection on weight stigma in healthcare using resources from Rudd Center for Food Policy & Health. *Weight stigma in healthcare.* University of Connecticut. https://supportiveobesitycare.rudd.center.uconn.edu/resources/*(Feedback from the Program Director)*
Pathophysiology	e-Lecture: • Neurohormonal Pathways of Obesity *(*b*Post lecture quiz)*	Chapters from Handbook of Obesity – Volume 1;4th ed: • Lynes MD et al. Bioenergetics and obesity. (pp. 69–78). • Ying T et al. The role of adipocyte precursors in development and obesity. (pp. 148–154). • Grobe JL et al. CNS regulation of energy balance. (pp. 207–215). • Kairupan TS et al. Gastrointestinal regulation of energy balance. (pp. 216–224). • Martinez-Guryn K et al. Gut microbiome and obesity. (pp. 225–232). • Lambert E. Sympathetic nervous system and obesity. (pp. 233–240). • Purnell JQ et al. Hypothalamic–pituitary hormones and obesity. (pp. 241–249). • Kajimura S. Brown, beige, and white adipocyte development. (pp. 250–258). • Samms RJ et al. Adipose tissue metabolism, adipokines, and obesity. (pp. 259–266). *(*b*Discussion with Program Director)*	–	–
Body Weight Regulation & Body Composition	e-Lectures: • Body Composition Part 1: Introduction and anthropometric Methods • Body Composition Part 2: Criterion Methods • Assessment of Energy Expenditure & Physical Activity *(*b*Post lecture quiz)*	Chapters from Handbook of Obesity – Volume1;4th ed: • Heymsfield S et al. Measurement of total adiposity, regional fat depots, and ectopic fat. (pp. 28–37). • Schutz Y et al. Resting metabolic rate, thermic effect of food, and obesity. (pp. 286–295). • Ross R et al. Energy cost of exercise, post-exercise metabolic rates, and obesity. (pp. 296–311). ^d^Chapter from Handbook of Obesity – Volume2;5th ed • Neeland I et al. Indicators of central adiposity. (pp. 99–114) Additional Readings: • Ainsworth BE et al. (2015). The current state of physical activity assessment tools. *Progress in Cardiovascular Diseases, 57*(4), 387–395. • Delsoglio M et al. Indirect Calorimetry in Clinical Practice. *J Clin Med*. 2019; 8:1387. *(*b*Discussion with Program Director)*	Conducts indirect calorimetry and bioelectrical impedance analysis (*Direct observation by obesity medicine faculty)*	–
Metabolic & Biomechanical Conditions & Complications	e-Lectures: • Polycystic Ovary Syndrome and Infertility • Metabolism Dysfunction-Associated Steatotic Liver Disease *(*b*Post lecture quiz)*	*Chapters from Handbook of Obesity – Volume1;4*th *ed:* • Sanchez AM et al. Obesity and heart disease. (pp. 461–468). • Hall JE et al. Obesity and hypertension. (pp. 469–480). • Chiu S et al. Obesity and lipoprotein metabolism. (pp. 481–487). • Kirwan JP et al. Obesity and insulin resistance. (pp. 488–495). • Stefan N. Obesity and type 2 diabetes. (pp. 496–502). • Patel AP et al. Obesity and cancer. (pp.511-518). • Demir M et al. Obesity and liver diseases. (pp. 537–547). • Camilleri M et al. Obesity, gallbladder, and gastrointestinal diseases. (pp. 527–536). • Mediwala S et al. Obesity, osteoarthritis, and bone disorders. (pp. 565–571). • Dixon AE et al. Obesity, lung function, and lung disease. (pp. 548–555). • Swerdloff R et al. Obesity and reproductive dysfunction. (pp. 588–594). Additional Readings: • Mollan SP et al. Idiopathic intracranial hypertension: Consensus guidelines on management. J Neurol Neurosurg Psychiatry. 2018; 89:1088–1100. *(*b*Discussion with Program Director)*	Longitudinal obesity medicine clinic (*OMEC based milestone evaluation and Mini-CEX by obesity medicine faculty)* Multidisciplinary subspecialty rotations (up to 4 selected): • Reproductive Endocrine and Infertility • Sleep Medicine • Hepatology • Preventive Cardiology and Lipid • Endocrinology • Pediatric Weight Management • Sleep Therapy • Menopause *(*b*Preceptor evaluations)*	eSupplemental readings: • Nagappa M et al. Validation of the STOP-Bang Questionnaire as a Screening Tool for Obstructive Sleep Apnea among Different Populations: A Systematic Review and Meta-Analysis. PLoS One. 2015 Dec 14; 10(12):e0143697. • European Association for the Study of the Liver; European Association for the Study of Diabetes; European Association for the Study of Obesity. EASL-EASD-EASO Clinical Practice Guidelines on the management of metabolic dysfunction-associated steatotic liver disease (MASLD): Executive Summary. Diabetologia. 2024 Nov; 67(11):2375-2392 • Rinella ME et al. AASLD Practice Guidance on the clinical assessment and management of nonalcoholic fatty liver disease. Hepatology. 2023 May 1; 77(5):1797–1835. • Teede HJ et al. International PCOS Network. Recommendations from the 2023 international evidence-based guideline for the assessment and management of polycystic ovary syndrome. Eur J Endocrinol. 2023 Aug 2; 189(2):G43-G64.
Psychological Conditions & Complications	e-Lecture: • Strategies for Assessing and Managing Binge Eating Behavior *(*b*Post lecture quiz)*	*Chapters from Handbook of Obesity – Volume1;4*th *ed:* • Mond JM et al. Eating disorders and obesity. (pp. 384–391). • Melamed OC et al. Obesity, Mental Health, and Health-Related Quality of Life. (pp. 581–587). *(*b*Discussion with Program Director)*	Longitudinal obesity medicine clinic (*OMEC based milestone evaluation and Mini-CEX by obesity medicine faculty)* Required interprofessional rotation with bariatric psychology (b*Preceptor evaluation)*	Supplemental Reading: • Morgan JF et al. The SCOFF questionnaire: a new screening tool for eating disorders. West J Med. 2000 Mar; 172(3):164-5. Logging patient cases that address: • Binge eating • Night eating (*Review cases with fellowship director)*
Secondary Obesity	e-Lectures: • Weight gain promoting Medications • Emerging from the shadows: Recognizing and treating lipedema OMA Academy. • Secondary Obesity: Exploring Endocrine and Rare Adipose Tissue Disorders *(*b*Post lecture quiz)*	Chapters from Handbook of Obesity – Volume2;5th ed: • Ameer, B. et al. Drug-associated weight gain and clinical alternatives. (pp. 269–280). Additional Readings: • Nieman LK et al. The Diagnosis of Cushing's Syndrome: An Endocrine Society Clinical Practice Guideline. J Clin Endocrinol Metab. 2008; 93:1526–40 *(*b*Discussion with Program Director)*	Longitudinal obesity medicine clinic (*OMEC based milestone evaluation and Mini-CEX by obesity medicine faculty)*	Reflection Assignments: The fellow is expected to review current literature on HIV medications and weight management for individuals with HIV and then respond to assignment questions based on their findings. *(Feedback from the Program Director)* Additional Resources: • Unbound Medicine, Inc. (2020). Johns Hopkins Menopause Guide 17+: Improving Women's Health [Mobile application]. Logging patient cases that address: • ∗Secondary obesity • bLipedema (*Review cases with fellowship director)*
Genetic & Syndromic Obesity	e-Lecture: • Genetic Disorders Associated with Childhood Obesity *(*b*Post lecture quiz)*	Chapters from Handbook of Obesity – Volume1;4th ed: • Mosbah H et al. Single-gene defects and obesity. (pp. 123–132). Chapters from Handbook of Obesity – Volume2;5th ed: • Farooqi S et al. Genetic Obesity Syndromes. pp.123-129 • Han J. et al. Treatment of Rare Forms of Genetic Obesity. (pp 402–411).	Required multidisciplinary rotation in pediatric obesity medicine *(*b*Preceptor evaluation)*	Logging patient cases that address: • bGenetic obesity (*Review cases with fellowship director)*
Initial Obesity Medicine Evaluation & Assessment	–	Chapters from Handbook of Obesity – Volume2;5th ed: • Kushner RF et al. Classification, evaluation, and staging of the patient with obesity. (pp 99–112). Additional Readings: • Kushner RF et al. Weight History in Clinical Practice: The State of the Science and Future Directions. Obesity (Silver Spring). 2020 Jan; 28(1):9–17. • Donini LM et al. Definition and Diagnostic Criteria for Sarcopenic Obesity: ESPEN and EASO Consensus Statement. Obes Facts. 2022; 15(3):321–335. Epub 2022 Feb 23. *(*b*Discussion with the Program Director)*	Longitudinal obesity medicine clinic (*OMEC based milestone evaluation and Mini-CEX by obesity medicine faculty)*	-
Nutrition	e-Lecture: • Meal Plans for Weight Loss *(*b*Post lecture quiz)*	Chapters from Handbook of Obesity – Volume1;4th ed: • Heilbronn L et al. Biology of Calorie Restriction and Intermittent Fasting. (pp. 95–103). • Cheon E et al. Obesity and dietary intake. (pp. 331–338). • Zheng M et al. Beverages and obesity: A review of current scientific evidence. (pp. 339–346). • Binks M. The role of the food industry in obesity. (pp. 395–405). Chapters from Handbook of Obesity – Volume2;5th ed. • Hall KD. Diet composition and weight loss: Is a calorie a calorie? (pp. 177–181). • Manoogian ENC et al. Circadian rhythms and weight loss: Timed food intake. (pp. 182–196). • Dinu M et al. Comparison of modern-millennial diets. (pp. 197–204). • Zuraikat FM et al. Dietary energy density and its contribution to weight control. (pp. 205–214). • Champagne CM. Dietary patterns as a basis for diet selection. (pp. 215–224). • Thom G et al. Personalized and precision nutrition in the management of obesity: Can we tailor the treatment to the patient? (pp. 258–268). *(*b*Discussion with the Program Director)*	Longitudinal obesity medicine clinic (*OMEC based milestone evaluation and Mini-CEX by obesity medicine faculty)* Required multidisciplinary subspecialty rotation in nutrition *(*b*Preceptor evaluation)*	Supplemental readings: • Estruch R et al. (2018). Primary prevention of cardiovascular disease with a Mediterranean diet supplemented with extra-virgin olive oil or nuts. *The New England Journal of Medicine, 378*(25), e34. • Appel L et al. (1997). A clinical trial of the effects of dietary patterns on blood pressure. The New England Journal of Medicine, 336(16), 1117–1124. • Gudzune KA et al. Efficacy of commercial weight-loss programs: an updated systematic review. Ann Intern Med. 2015 Apr 7; 162(7):501-12. Logging patient encounters: The fellow will be required to log patient encounters which address the following case topics or diagnoses: • Dietary Tracking • DASH diet • Mediterranean diet • Low-Carb diet • Meal replacement • Intermittent fasting • Vegetarian/Vegan (*Review cases with fellowship director)* Role Play Session: The fellow is required to conduct a practice session on developing a personalized nutrition plan, including counseling patients based on their medical needs, cultural preferences, dietary habits, and readiness for change. The session focuses on goal-setting, behavior change strategies, and aligning recommendations with evidence-based guidelines for obesity and metabolic diseases
Physical Activity	e-Lectures: • Fitness Assessments and Exercise Counseling. • Exercise Programming and Physical Activity Considerations for Patients with Obesity and Chronic Pain *(*b*Post lecture quiz)*	Chapters from Handbook of Obesity – Volume1;4th ed: • Biddle SJH et al. Obesity and sedentary time at work and home. (pp. 347–355). • Clina JG et al. Leisure time physical activity and obesity. (pp. 356–362). Chapters from Handbook of Obesity – Volume2;5th ed: • Jakicic JM et al. Physical activity and weight loss: Volume, intensity, and mode. (pp. 225–232). • Gibbs BB. Modifying sedentary behavior for the prevention and treatment of obesity. (pp. 233–240). *(*b*Discussion with the Program Director)*	Longitudinal obesity medicine clinic (*OMEC based milestone evaluation and Mini-CEX by obesity medicine faculty* Required interprofessional rotation in exercise physiology or physical therapy *(*b*Preceptor evaluation)*	Role Play Session: The fellow is required to conduct a practice session on setting up a personalized physical activity plan, including counseling patients while considering medical limitations, fitness level, lifestyle factors, and motivational barriers. The session emphasizes goal-setting, gradual progression, and culturally appropriate recommendations to support long-term adherence.
Behavior Change	e-Lecture: • Self-Monitoring for Weight Management. *(*b*Post lecture quiz)*	Chapters from Handbook of Obesity – Volume1;4th ed: • Fuemmeler BF et al. Tobacco use, marijuana use, vaping, and obesity. (pp. 371–377). • Cerolini S et al. Sleep and obesity. (pp. 378–383). Chapters from Handbook of Obesity – Volume2;5th ed: • Tronieri JS et al. In-person behavioral approaches for weight management. (pp. 149–159). • Höchsmann C et al. Behavioral interventions delivered remotely. (pp. 160–167). • Newton RL et al. Cultural considerations in behavioral therapy for the management of obesity. (pp. 168–176). *(*b*Discussion with the Program Director)*	Longitudinal obesity medicine clinic (*OMEC based milestone evaluation and Mini-CEX by obesity medicine faculty)*	Workshop training: The Johns Hopkins Patient Engagement Program (PEP). PEP is a comprehensive, evidence-based communication training program that focuses on improving health care professionals' communication and relationship-building skills.
Pharmacotherapy	e-Lectures: • Sympathomimetic Amines • Lipase Inhibitors • Naltrexone SR/Bupropion SR • Phentermine/Topiramate ER • Glucagon-like peptide-1 (GLP1) receptor agonists • GIP and GLP-1 receptor dual agonists • Leptin, Lisdexamfetamine and Setmelanotide *(*b*Post lecture quiz)*	Chapters from Handbook of Obesity – Volume2;5th ed: • Burak MF et al. Mechanisms of drug action in obesity. (pp. 281–290). • Magkos F. Metabolic effects of progressive weight loss. (pp. 291–300). • Greenway FL et al. Older drugs used to manage obesity: Sympathomimetics and orlistat. (pp. 301–311). • Serlie MJ. Serotonergic drugs for treating obesity. (pp. 312–317). • Perdomo CM et al. Antidiabetic drugs that reduce weight and are cardioprotective. (pp. 318–332). • Garvey WT. Approved combination agents: Phentermine/Topiramate. (pp. 333–347). • Onakpoya IJ et al. Approved combination agents: Naltrexone-Bupropion. (pp. 348–366). • Asswad RG et al. Drugs that affect body weight: The glucagon-like peptide-1 agonists. (pp. 367–375). • Greenway FL. Hydrogels in the treatment of obesity. (pp. 376–383). • Min T et al. Dual and triple-action peptides. pp. 384–393. • Barenbaum SR et al. Antiobesity medications on the horizon. (pp. 394–401). • Moore JM et al. Special issues in using medications in children and adolescents. pp. (412–428). • Haggans CJ et al. Dietary supplements and weight management. (pp. 429–440). *(*b*Discussion with the Program Director)*	Longitudinal obesity medicine clinic (*OMEC based milestone evaluation and Mini-CEX by obesity medicine faculty)*	Supplemental readings: • Wilding JPH, STEP 1 Study Group. Once-Weekly Semaglutide in Adults with Overweight or Obesity. N Engl J Med. 2021 Mar 18; 384(11):989–1002. • Jastreboff AM et al.; SURMOUNT-1 Investigators. Tirzepatide Once Weekly for the Treatment of Obesity. N Engl J Med. 2022 Jul 21; 387(3):205–216. • Lincoff AM et al. SELECT Trial Investigators. Semaglutide and Cardiovascular Outcomes in Obesity without Diabetes. N Engl J Med. 2023 Dec 14; 389(24):2221–2232. • Lincoff AM et al. SELECT Trial Investigators. Semaglutide and Cardiovascular Outcomes in Obesity without Diabetes. N Engl J Med. 2023 Dec 14; 389(24):2221–2232. • Aronne, L. J. et al. Continued treatment with tirzepatide for maintenance of weight reduction in adults with obesity: The SURMOUNT-4 randomized clinical trial. *JAMA*, 331(1), 38–48. (2024). • Wadden TA et al. Liraglutide 3.0 mg and Intensive Behavioral Therapy (IBT) for Obesity in Primary Care: The SCALE IBT Randomized Controlled Trial. Obesity (Silver Spring). 2020 Mar; 28(3):529-536 • Aronne LJ et al. Evaluation of phentermine and topiramate versus phentermine/topiramate extended-release in obese adults. Obesity (Silver Spring). 2013 Nov; 21(11):2163-71. • Allison DB et al. Controlled-release phentermine/topiramate in severely obese adults: a randomized controlled trial (EQUIP). Obesity (Silver Spring). 2012 Feb; 20(2):330-42 • Apovian CM, COR-II Study Group. A randomized, phase 3 trial of naltrexone SR/bupropion SR on weight and obesity-related risk factors (COR-II). Obesity (Silver Spring). 2013 May; 21(5):935-43. • Torgerson JS et al. XENical in the prevention of diabetes in obese subjects (XENDOS) study: a randomized study of orlistat as an adjunct to lifestyle changes for the prevention of type 2 diabetes in obese patients. Diabetes Care. 2004 Jan; 27(1):155-61. Role Play Session: The fellow is required to conduct a practice session on counseling for medications used in obesity management, including discussing indications, expected outcomes, side effects, and cost or access considerations, and engaging the patient in shared decision-making.
Non-Surgical Procedures	e-Lectures: • Endoscopic Therapies for Obesity Part 1 & 2 • Bariatric Arterial Embolization *(*b*Post lecture quiz)*	Chapters from Handbook of Obesity – Volume2;5th ed: • Roberts CF et al. Other bariatric procedures: Nerve stimulation, gastric balloons, intestinal liners, and others. (pp. 501–510).	Required multidisciplinary rotation in bariatric endoscopy with gastroenterologist *(*b*Preceptor evaluation)*	Logging patient encounters: The fellow will be required to log patient encounters which address the following case topics or diagnoses: • Endoscopic sleeve (*Review cases with fellowship director)*
Surgical Procedures	e-Lecture: • Bariatric Surgery: Indications, Post-Surgery Nutrition and Follow-up *(*b*Post lecture quiz)*	Chapters from Handbook of Obesity – Volume2;5th ed: • Buchwald H. Historical overview of metabolic and bariatric surgery. (pp. 447–456). • Ross RC et al. Overview: Current metabolic operations and long-term outcomes. (pp. 457–467). • Roser P et al. Effects of metabolic surgery on type 2 diabetes. (pp. 468–475). • Tan WH et al. Cardiovascular outcomes in metabolic surgery. (pp. 476–484). • Brown JC et al. Effect of bariatric surgery on cancer. (pp. 485–492). • Ogle S et al. Bariatric surgery in pediatric patients. (pp. 493–500). *(*b*Discussion with the Program Director)*	Required multidisciplinary rotation in metabolic-bariatric surgery with surgeons including sessions in operating room and inpatient setting *(*b*Preceptor evaluation)*	Supplemental readings: • Mechanick, JI et al. Clinical practice guidelines for the perioperative nutrition, metabolic, and nonsurgical support of patients undergoing bariatric procedures – 2019 update: Cosponsored by American Association of Clinical Endocrinologists/American College of Endocrinology, The Obesity Society, American Society for Metabolic & Bariatric Surgery, Obesity Medicine Association, and American Society of Anesthesiologists – Executive summary. Endocr Pract. 2019 Dec; 25(12):1346–1359. • Eisenberg D et al., 2022 American Society for Metabolic and Bariatric Surgery (ASMBS) and International Federation for the Surgery of Obesity and Metabolic Disorders (IFSO): Indications for Metabolic and Bariatric Surgery. Surg Obes Relat Dis. 2022 Dec; 18(12):1345–1356. *Logging patient encounters:* The fellow will be required to log patient encounters which address the following case topics or diagnoses: • Roux-en-Y Gastric Bypass, • Sleeve Gastrectomy, • Bariatric Surgery Revisions • Biliopancreatic Diversion with Duodenal Switch • Single Anastomosis Duodenal-Ileal Switch (Review cases with fellowship director) *Role Play Session:* • The fellow is required to conduct a practice session on counseling for surgical procedures, including explaining bariatric surgery options, discussing risks and benefits, setting appropriate expectations, addressing patient concerns, and facilitating shared decision-making tailored to each patient's medical and cultural context.
Multicomponent Obesity Medicine Treatment Plan	e-Lecture: • Obesity Management in Pediatrics *(*b*Post lecture quiz)*	• Garvey WT et al.; Reviewers of the AACE/ACE Obesity Clinical Practice Guidelines. American Association of Clinical Endocrinologists and American College of Endocrinology comprehensive clinical practice guidelines for medical care of patients with obesity. Endocrine Practice. 2016 Jul; 22(Suppl 3):1–203. • LeBlanc ES et al. Behavioral and Pharmacotherapy Weight Loss Interventions to Prevent Obesity-Related Morbidity and Mortality in Adults: Updated Evidence Report and Systematic Review for the US Preventive Services Task Force. JAMA. 2018 Sep 18; 320(11):1172–1191. • Hampl SE et al. Clinical Practice Guideline for the Evaluation and Treatment of Children and Adolescents With Obesity. Pediatrics. 2023 Feb 1; 151(2):e2022060640. Erratum in: Pediatrics. 2024 Jan 1; 153(1):e2023064612. *(*b*Discussion with the Program Director)*	Longitudinal obesity medicine clinic (*OMEC based milestone evaluation and Mini-CEX by obesity medicine faculty)* Longitudinal obesity medicine group visits (*OMEC based milestone evaluation and Mini-CEX by obesity medicine faculty)* Required multidisciplinary rotation in pediatric obesity medicine *(*b*Preceptor evaluation)*	Reflection Assignments: The fellow reviews the guidelines from AACE/ACE, TOS/AHA/ACC, and USPSTF on obesity prevention and write a reflection piece to compare the recommendations from these guidelines. *(Reviewed by the program director)* Logging patient encounters: The fellow is required to log patient encounters which address the following case topics or diagnoses: • Pharmacotherapy (any cases with pharmacotherapy use) • Bariatric surgery/procedures (*Review cases with fellowship director)* Role Play Session*:* Culturally specific counseling practice session addressing dietary practices, religious observances, health beliefs, and language or literacy barriers.
Longitudinal Obesity Medicine Care	–	Chapters from Handbook of Obesity – Volume2;5th ed: • Anton SD et al. Preventing weight regain after weight loss. (pp. 241–257). *(*b*Discussion with the Program Director)*	Longitudinal obesity medicine clinic (*OMEC based milestone evaluation and Mini-CEX by obesity medicine faculty)*	Supplemental reading: Varkevisser RDM et al. Determinants of weight loss maintenance: a systematic review. Obes Rev. 2019 Feb; 20(2):171–211.
Health Systems & Population Health	–	Chapters from Handbook of Obesity – Volume1;4th ed: • Peterman JE et al. Transportation policies and obesity. (pp. 406–414). • White KR et al. Urban environment and obesity. (pp. 415–422). Chapters from Handbook of Obesity – Volume2;5th ed: • Broyles ST et al. Re-engineering the built environment: Progress in schools, worksites, neighborhoods, and communities. (pp. 39–49). • Biener A et al. The economic costs of obesity. (pp. 50–58). • Ananthapavan J et al. Cost-effectiveness of obesity prevention and treatment. (pp. 59–78). • Barquera S et al. Role of government and non-government organizations in the obesity pandemic and its prevention. (pp. 79–87). • Anekwe CV et al. Role of reimbursement in the delivery of treatment for obesity in adults and children. (pp. 88–98). *(*b*Discussion with the Program Director)*	–	Training for medical billing for obesity medicine and chart review by a billing expert after one month of billing. Contribution to an online educational module on obesity distributed nationally and lead discussions at the monthly Obesity Medicine Rounds. Serving as a small group facilitator for first-year medical students in the Nutrition and Behavior course.
Public Health & Prevention	e-Lectures: • Obesity Surveillance and Epidemiology • Prevention of Obesity *(*b*Post lecture quiz)*	Chapters from Handbook of Obesity – Volume1;4th ed: • Seidell JC. Worldwide and regional prevalence of obesity. (pp. 48–52). • Park S et al. Age, sex, ethnicity, and other sources of variation in the prevalence of obesity. (pp. 53–63). Chapters from Handbook of Obesity – Volume2;5th ed: • Andrews P et al. Dietary changes in human evolution: Implications for the prevention of obesity. (pp. 3–10). • Pontzer H. Evolutionary changes in physical activity, diet, and energy expenditure: Implications for the prevention of obesity. (pp. 11–17). • Phelan S. Prevention of obesity in adults. (pp. 18–27). • Lobstein T et al. The prevention of obesity in children and adolescents. (pp. 28–38). *(*b*Discussion with the Program Director)*	–	Reflection Assignments: After completing the Prevention of Obesity e-module, the fellow is required to write a reflection piece that describes the clinical application of primary, secondary, and tertiary interventions in obesity management. *(Reviewed by the Program Director)*
Professional Development & Identity as an Obesity Medicine Physician	–	–	Longitudinal obesity medicine clinic (*OMEC based milestone evaluation and Mini-CEX by obesity medicine faculty)* Self-evaluation at baseline and 6 months Faculty evaluations of the fellow at baseline, 6 and 9 months and 12 months if needed	Half-day per week for research, focusing on scholarly activity and manuscript publication. Participation in monthly Obesity Medicine Fellowship Council meetings and a weekly diabetes and obesity journal club. Quarterly meetings with the program director to address topics (e.g., CV, NIH biosketch, scientific writing).

Abbreviations: OMEC—Obesity Medicine Education Collaborative Competencies; Mini-CEX—mini clinical evaluation exercise; OMA—Obesity Medical Association.

a Other instructional activities include directed readings, workshops, role-play activities, and reflections.

b Plans for future integration at the Johns Hopkins Obesity Medicine Fellowship.

c Handbook of Obesity - Volume 1: Epidemiology, Etiology, and Physiopathology (4th ed.), Edited by G. A. Bray & C. Bouchard, CRC Press, 2023.

d Handbook of Obesity - Volume 2: Clinical Applications (5th ed.), Edited by G. A. Bray & C. Bouchard, CRC Press, 2024.

e Required Readings are essential materials that all fellows must read to meet the core learning objectives. Supplemental Readings are optional resources provided to enhance understanding or explore topics in greater depth, supporting differentiated learning.

In general, e-lectures and directed readings aim to establish foundational knowledge, whereas clinical experiences in longitudinal obesity medicine clinic as well as multidisciplinary and interprofessional rotations facilitate knowledge and skill application through hands-on practice or direct observation. Didactics and directed readings are also timed, as feasible, to coincide with relevant clinical experiences. For example, the assignment of the metabolic-bariatric surgery e-lecture is aligned with a clinical rotation in this field to ensure immediate application of knowledge in patient care settings.

Three additional types of educational activities are included to further support the development of knowledge as well as communication skills, clinical decision-making, and documentation proficiency. 1) Reflection assignments are structured learning activities designed to deepen critical thinking and promote synthesis of current evidence into clinical practice. Fellows are expected to engage with recent literature on specific topics, such as obesity treatment in individuals with HIV, and respond to targeted assignment questions that require analysis, application, and integration of their findings. These assignments encourage fellows to critically appraise new research, consider its relevance to patient care, and strengthen their ability to translate evidence into clinical decision-making. 2) Role play sessions are strategically integrated at the end of the first month of training to enhance practical communication skills. Fellows engage in simulated counseling activities for surgical procedures, pharmacotherapy management, and culturally sensitive interventions, as well as the development of personalized nutrition and physical activity plans. 3) Fellows complete training in motivational interviewing through the Johns Hopkins Patient Engagement Program, gaining evidence-based communication skills during an intensive workshop facilitated by trained psychologists that enhance patient-physician relationships.

Our fellowship curriculum places significant emphasis on research, scholarly activity, and professional development as core elements. Fellows are provided with mentorship and dedicated time for research, manuscript writing, and scholarly projects. Professional development activities include medical billing training with expert feedback, curriculum vitae and biosketch development, and scientific writing workshops. The fellow has opportunities to develop their skills as a medical educator through contributions to the Johns Hopkins Physician Education and Assessment Center module – an online educational platform designed to enhance resident obesity medicine education nationwide – and opportunities to facilitate small group discussions on nutrition, behavior change and obesity for medical students. Finally, the fellowship promotes peer learning through hosting monthly Obesity Medicine Rounds, which is led by the fellow. These sessions provide opportunities to explore topics of personal interest beyond the standard curriculum and network with experts across the institution and beyond, fostering professional growth and collaboration.

**Fig. 1:**
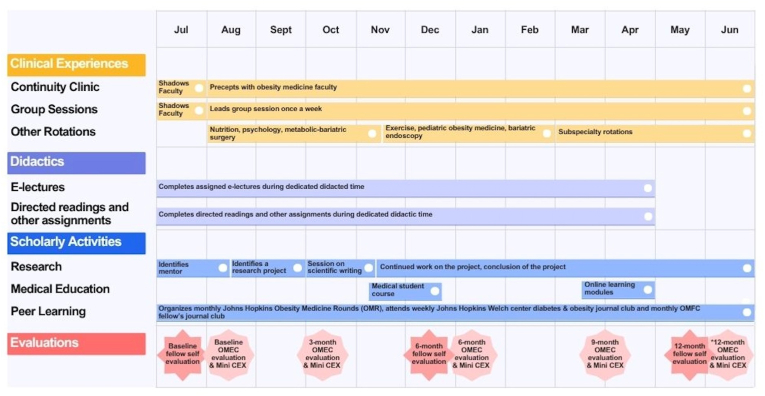
**Legend. Timeline of Obesity Medicine Fellowship Activities.** This figure presents the structured, year-long timeline of core activities within the Obesity Medicine Fellowship program, organized across four domains: Clinical Experiences, Didactics, Scholarly Activities, and Evaluations. Clinical Experiences include ongoing continuity clinic and group session facilitation, as well as rotations in nutrition, psychology, pediatric obesity medicine, bariatric endoscopy, metabolic-bariatric surgery, and other subspecialties. Didactic components consist of e-lectures and directed readings completed during protected time. Scholarly Activities encompass research project development, engagement in medical education (e.g., teaching a medical student course), and peer learning through journal clubs and obesity medicine rounds. Evaluations include fellow self-assessments and faculty assessments at baseline, 3, 6, 9, and 12∗ months using OMEC competencies, along with mini-CEX exercises to assess clinical skills. Activities are sequenced to support progressive development of clinical and academic competencies over the fellowship year. Abbreviations: OMFC – Obesity Medicine Fellowship Council; OMEC – Obesity Medicine Education Collaborative; Mini-CEX–Mini-clinical evaluation exercise ∗12-month evaluation conducted if needed.

The overall structure and sequencing of the fellowship's clinical, didactic, scholarly, and evaluation activities and implementation are summarized in Fig. 1, which illustrates the longitudinal integration of core curriculum components throughout the academic year. Fellows initially provide patient care under direct faculty supervision, gradually increasing their independence as skills development. Training includes one-on-one faculty precepting sessions at the end of each clinical session, which allow fellows to apply and clarify their knowledge under the guidance of an obesity medicine physician. The fellow spends approximately 60 % of their time in an obesity medicine continuity clinic (5-half day sessions/week) and 40 % on didactics, multidisciplinary and interprofessional core rotations, subspecialty elective rotations, and scholarly activities (including research, teaching, meetings, and conferences).

### 2.5 Step 5: implementation

Implementation requires a structured, developmental process that unfolds across four key stages: generating internal and external support, planning for change through the development of clearly defined goals, learning objectives, and educational strategies, operationalizing implementation, and ensuring long-term viability. Based on these stages, we used a comprehensive approach recommended by Kern et al. to guide the successful launch and long-term sustainability of the training program [12].

#### 2.5.1 Identifying resources

The implementation of our curriculum began with identifying key resources. We identified core and associate faculty, and a program coordinator was appointed. Protected time was allocated for the program director and core faculty to develop the curriculum. Physical space was secured within the existing clinical infrastructure. We identified institutional educational platforms to deliver curricular content, generate the fellow's schedule, conduct evaluations and assessments, and track clinical hours.

#### 2.5.2 Obtaining support

We obtained internal support from the Graduate Medical Education Committee, the Department of Medicine, and the Division of General Internal Medicine leadership, with the Division serving as the program's official sponsor. In alignment with OMFC requirements [21], the Program Director is an ABOM Diplomate. All other core faculty also hold ABOM certification and have backgrounds in internal medicine or medicine-pediatrics. In addition to the core faculty, key supporters include a multidisciplinary team encompassing nutrition, exercise physiology/physical therapy, pediatric obesity medicine, metabolic-bariatric surgery, bariatric endoscopy, and bariatric psychology. A grant from the OMFC provided some financial support, which funded the development of curriculum materials and evaluation tools as well as administrative costs for the first several years. To broaden awareness and build external support, we conducted outreach to regional residency program directors and relevant stakeholders to raise awareness of the new fellowship.

#### 2.5.3 Developing administrative mechanisms to support the curriculum

We established an administrative structure to define roles and responsibilities among the core faculty, including formally establishing the obesity medicine education committee, the clinical competency committee (CCC) and program evaluation committee (PEC). The obesity medicine education committee has served as the central body for guiding curriculum planning and implementation. The committee met regularly to define the goals and objectives of the curriculum, oversee its development, and ensure alignment across educational strategies, curricular content, clinical experiences, and evaluation methods. The program aimed for the CCC to meet quarterly to review and assess the progress of fellow's clinical skill development, while the PEC would meet at least annually to review the progress of the fellowship program and identify areas for improvement. The CCC would be comprised of all core obesity medicine faculty and other associate faculty could be invited, as needed. The PEC would be comprised of the obesity medicine fellow, core and associate obesity medicine faculty, as well as external obesity medicine physicians.

#### 2.5.4 Anticipating and Addressing barriers

We identified that establishing a consistent pipeline of qualified trainees might be a challenge in implementing the fellowship program. Therefore, early and sustained outreach efforts targeting internal medicine and family medicine residency program directors was employed. Core faculty played a central role in raising the program's visibility through active involvement in obesity medicine electives for residents and medical student education, as well as curated interest with individual trainees through mentorship in research and other scholarly projects. These efforts were instrumental in building trust, generating interest, and securing trainee buy-in within the institution, ultimately strengthening the program's visibility and supporting ongoing recruitment efforts.

#### 2.5.5 Pilot, rollout, and full implementation

The curricular content was piloted with feedback from prior learners to ensure alignment with learner needs. These learners have included preventive medicine residents, medicine-pediatrics residents, and general internal medicine fellows in the clinical research track, all of whom completed their weekly continuity clinics within the obesity medicine clinical practice at Johns Hopkins. The obesity medicine fellowship began in August 2021 with one obesity medicine fellow and was open to applicants who had completed residency training in internal medicine, family medicine, or medicine-pediatrics. We created a fellowship manual that outlines didactic content and provides a timeline to ensure the fellow reviews foundational topics essential for obesity medicine practice during the first quarter of the academic year.

In the first month, the fellow focuses primarily on structured learning, including e-lectures, directed readings, reflection assignments, role-playing exercises, and motivational interviewing training. The fellow also observes obesity medicine clinical encounters with faculty and begins participating in patient care. After this first month, the fellow begins delivering obesity medicine care under supervision, which is precepted by obesity medicine faculty members. Fellows transition from direct to indirect supervision over time as supported by CCC assessments of their clinical competence. The fellow builds a panel of patients and reviews each case with an assigned faculty preceptor. The fellow will also complete core multidisciplinary rotations (i.e., metabolic-bariatric surgery, bariatric endoscopy, and pediatric obesity medicine) and core interprofessional rotations (i.e., nutrition, psychology, exercise and physical therapy). During the final four months, the fellow may pursue subspecialty elective rotations to broaden their expertise in obesity-related care. These elective rotations include sleep medicine, reproductive endocrinology and infertility, hepatology, preventive cardiology, lipidology, endocrinology, and menopause. Fellows with training in internal medicine-pediatrics or family medicine may also complete an additional four-week rotation in pediatric obesity medicine.

At the beginning of training, fellows initiate the process of identifying a faculty mentor and selecting a research or other scholarly project aligned with their interests. Early in the fellowship year, fellows participate in a dedicated session on scientific writing to support the development of their project. Under the guidance of their mentor, fellows continue to refine and advance their work throughout the year, with regular check-ins to ensure progress. By the end of the training period, fellows are expected to complete and present their project, with the goal of contributing to scholarly dissemination through abstracts, presentations, or manuscript submissions.

#### 2.5.6 Curriculum enhancement and maintenance

Since its launch, the curriculum has undergone iterative refinement over successive cycles. Feedback from both fellows and faculty has guided ongoing revisions, ensuring that the curriculum remains aligned with its educational goals and responsive to emerging best practices in obesity medicine. These continuous improvements have helped sustain the curriculum's relevance and effectiveness. Further details are provided in Section 2.6.

### 2.6 Step 6: Evaluation and feedback

Evaluation and feedback are integral to both the assessment of individual learners and the continuous improvement of the fellowship program and curriculum. In this section, we describe our approach to evaluating fellows as well as mechanisms for overall program evaluation.

#### 2.6.1 Individual assessments of fellows

The curriculum emphasizes formative evaluation as an ongoing process to provide timely feedback and guide continuous improvement. Faculty complete milestone-based evaluations using OMEC competencies and assess clinical performance through tools like mini-clinical evaluation exercises (mini-CEXs). Evaluations are documented and managed using an online platform. Monthly meetings with the program director and ongoing clinical precepting serve as additional methods for evaluating the fellow's progress.

Trainees are assessed using milestone-based evaluations aligned with the six core domains of the OMEC competencies: Patient Care and Procedural Skills, Medical Knowledge, Practice-Based Learning and Improvement, Interpersonal and Communication Skills, Professionalism, Systems-Based Practice [20] (example assessments are available in **Supplemental Materials 1**). An initial evaluation after completing the first month of fellowship by obesity medicine faculty establishes a baseline of each fellow's knowledge and clinical competency. Subsequent evaluations are conducted every three to four months (Fig. 1), with an additional end-of-year evaluation if needed. These assessments guide individualized precepting strategies and inform the selection of supplemental educational resources tailored to each fellow's needs. In addition to faculty assessments, fellows complete a baseline self-evaluation and repeat self-assessments every six months. These self-evaluations are also aligned with the OMEC competencies and serve as a tool for fellows to reflect on their progress.

Mini-CEXs provide real time observation and feedback on a trainee's clinical competency by obesity medicine faculty. We created several mini-CEXs for obesity medicine (initial evaluation, follow-up care, group visit), by adapting the ACGME Mini-CEX Rater Assessment Form [22] (example assessments are available in **Supplemental Materials 2**). The mini-CEX assesses fellows across key competencies, including clinical history-taking, physical examination, communication and professionalism, clinical judgment, documentation, billing, and overall competence. Fellows are rated on a 1–9 scale, with qualitative feedback provided to support their development in obesity medicine care. Mini-CEXs take place at month 1 for a baseline evaluation, and again at 4-month intervals. A final mini-CEX may be completed at the end of the fellowship program if needed.

The program director meets monthly with the fellow to review progress, assess didactic comprehension, and provide mentorship. These meetings, along with periodic knowledge checks and feedback from role play and reflective exercises, help reinforce learning and identify areas for improvement. Clinical precepting is central to training, with fellows working under direct and indirect supervision, receiving real-time feedback from an assigned faculty preceptor daily. Evaluation data are reviewed quarterly by the CCC, where faculty assessments are discussed and the committee reaches agreement on the fellow's competency level for each OMEC competency. Feedback is shared with the fellow, and progress is tracked over time to guide individualized learning plans.

#### 2.6.2 Program evaluation

Program evaluation is a continuous process designed to assess the quality, effectiveness, and relevance of the fellowship program and its curriculum. First, the obesity medicine education committee meets monthly to coordinate and oversee the educational activities of learners at all levels across the institution, including the obesity medicine fellowship program. These meetings serve as a collaborative forum to evaluate ongoing educational projects and plan future initiatives.

The PEC plays a central role in the ongoing quality improvement and oversight of the fellowship program. It meets at least annually to review all aspects of the curriculum, including its structure, educational activities, evaluation data, and progress on action items from the previous meeting's report, as documented in the Annual Program Evaluation Action Plan. We obtain direct feedback from fellows and faculty about the fellowship program, which is used by the PEC. Fellows evaluate the program towards the end of their fellowship year and are encouraged to provide feedback to the program director at any time to improve their training experience. Faculty evaluations of the program are also conducted annually. The PEC responsibilities include:

• Planning, developing, implementing, and evaluating educational activities
• Reviewing and updating competency-based curriculum goals and objectives
• Conducting annual reviews based on confidential fellow and faculty evaluations
• Ensuring compliance with graduate medical education and institutional standards, including duty hours
• Systematically reviewing all components of the curriculum, including clinical rotations, didactic sessions, scholarly activity, and conference participation

Progress is tracked through subsequent PEC meetings, fellow and faculty feedback, and follow-up actions to ensure accountability and sustained program advancement. The Annual Program Evaluation Action Plan is submitted to the appropriate institutional representative annually.

### 2.7 Curriculum maintenance and enhancement

We have approached curriculum maintenance and enhancement as an iterative process. Throughout the first year — and continuing annually — the curriculum has been refined based on feedback from fellows, faculty, and PEC recommendations. To date, key refinements include adjustments to the duration and sequencing of clinical rotations, updates to directed readings, and augmenting learning through reflection assignments and role play. These enhancements reflect evolving educational goals, learner needs, and faculty observations regarding clinical preparedness and content integration. Overall, informal feedback consistently shows high satisfaction with the fellowship experience.

Since the program's inception, fellow and faculty feedback have helped optimize the structure of core rotations. For example, the metabolic-bariatric surgery rotation was moved earlier in the training year to allow fellows more time to build confidence discussing procedural options with patients and delivering peri-operative care. In addition, subspecialty elective rotations have evolved in response to fellow feedback, and the program is currently exploring how to integrate experiences in obesity advocacy, community engagement, and policy-level interventions based on fellow interest in these areas.

Future directions for the fellowship program include enhancing the assessment of trainees’ comprehension of didactic content by introducing mechanisms such as post-lecture quizzes and structured discussions around e-lectures and directed readings. Ongoing plans also include refining learning objectives for both e-lectures and clinical rotations to ensure alignment with educational goals and emerging clinical evidence. Curriculum content will continue to be updated regularly, and new topics will be introduced to further enrich the trainee experience. The program remains committed to continuous improvement, with curriculum changes guided by data, feedback, and a shared mission to train competent, compassionate, and forward-thinking obesity medicine physicians.

## 3 Discussion

Our obesity medicine fellowship offers a comprehensive, multidisciplinary and interprofessional approach to obesity care, grounded in evidence-based practices and designed to address critical gaps in postgraduate physician training. The program has evolved through iterative improvements based on fellow and faculty feedback, resulting in meaningful enhancements.

For institutions seeking to establish an obesity medicine fellowship, the description of our curriculum development and fellowship program implementation may serve as a potential model and practical roadmap. Our experience underscores the importance of early investment in faculty development, structured curriculum planning, and continuous quality improvement. Key elements for success included leveraging existing clinical infrastructure and combining the resources from the ABOM, ACGME-I and OMEC to identify key domains for obesity medicine fellowship training. In addition, identifying ABOM-certified faculty may be particularly important for forthcoming fellowship programs. A recent national survey of U.S. family medicine residency program directors identified faculty development as a key need in expanding obesity education [23]. Therefore, ensuring ABOM-certified faculty is likely essential for launching obesity medicine fellowships.

A key challenge in developing this fellowship program was the lack of standards for obesity medicine fellowship training and the lack of published literature on obesity medicine fellowship training. In fact, a recent review by Fitch and colleagues identified the need for standardized training and certification in obesity medicine [24]. While OMFC provides national guidance, it is not an accrediting organization and does not enforce standards. This absence of accreditation may lead to variability in training quality, educational outcomes for fellows, and clinical outcomes for patients. Evaluating the impact of obesity medicine fellowship training will be critical for building the evidence base needed to demonstrate their value to healthcare systems and public health. As the field of obesity medicine continues to develop, there may be an increasing need for standards in fellowship training as well as accreditation of fellowship training programs.

## 4 Limitations

This report reflects the development, implementation, and early outcomes of a single obesity medicine fellowship program at a U.S. academic medical center. While our experience offers insights that may inform the development of similar programs elsewhere, the findings and processes may not be generalizable to institutions with differing resources, faculty capacity, or patient populations. In the absence of national standards or accreditation, there may be variability in how obesity medicine fellowships are structured, implemented, and evaluated. Future research is needed to establish benchmarks and best practices that ensure consistency in training quality across programs.

## 5 Conclusions

An obesity medicine fellowship program can be designed and implemented to address key training gaps in obesity care through a structured curriculum shaped by faculty and fellow input. Sharing our development process offers a roadmap for others seeking to establish similar programs.

## 6 Summary takeaway messages

• There are significant training gaps in obesity care for physicians and obesity medicine fellowship may help close these gaps.
• This report outlines a process for developing, implementing and refining an obesity medicine fellowship, which helps to build a shared knowledge base and may promote training standards across institutions.
• Academic centers across the country should consider developing obesity medicine fellowships to build a pipeline of skilled, evidence-based obesity medicine physicians.
• Future work should focus on evaluating the long-term impact of obesity medicine fellowships on clinical outcomes, health systems, and workforce readiness.

## Author contributions

ZWC, KAG, and SR conceptualized this work. All authors were involved with developing the obesity medicine curriculum. ZWC wrote the first draft. All authors reviewed, edited, and approved the final submission and publication.

## Disclosures

ZWC, ML and SR declare that they have no competing interests. During the conduct of this work, KAG was faculty at the Johns Hopkins School of Medicine. KAG is now employed by the American Board of Obesity Medicine Foundation and was previously employed by the American Board of Obesity Medicine. She has received personal fees as a conference speaker from the American College of Cardiology, the American Diabetes Association, and PRI-MED; personal fees for participation on advisory boards for Eli Lilly and Company and Novo Nordisk; and travel support from the American College of Cardiology, the American Diabetes Association, Eli Lilly and Company, and Novo Nordisk. She has received royalties from the Johns Hopkins ACG System. Her former institution (10.13039/100007880Johns Hopkins) received grant funding from 10.13039/501100004191Novo Nordisk.

## Ethical adherence

This work only involved review of description of our curriculum development process, and therefore, would not be considered human subjects research per U.S. Department of Health & Human Services definitions.

## Declaration of artificial intelligence (AI) and AI-assisted technologies

During the preparation of this work, the author(s) used artificial intelligence (AI) tools to enhance readability and improve language clarity. Specifically, AI was used for language refinement and editing suggestions. After using these tools, the author(s) reviewed and edited the content as needed and take(s) full responsibility for the content of the publication.

## Source of funding

Development of the Johns HopkinsObesity Medicine Fellowship was supported, in part, by a grant from the Obesity Medicine Fellowship Council (PI: Gudzune).
