# Supplementary material for: Developing and implementing an obesity medicine fellowship program: Experience at a U.S. academic medical center
Source: Obes Pillars. 2025 Aug 27;16:100204. doi: 10.1016/j.obpill.2025.100204 (PMC12424414; doi:10.1016/j.obpill.2025.100204)
Supplement: Multimedia component 1 [file mmc1.docx]

**Supplemental Materials. Developing and Implementing an Obesity Medicine Fellowship Program: Experience at a U.S. Academic Medical Center**

**Supplemental Materials 1:** Baseline Faculty Evaluation of Obesity Medicine Fellow**,** Program Evaluation by the Fellow and Faculty Evaluation of the Program

*Note:* The Baseline Obesity Medicine Fellow Self-Assessment, Quarterly Faculty Evaluations of the Fellow, End-of-Year Fellow Self-Assessment are available upon request from the corresponding author.

**Supplemental Materials 2:** Initial and Follow-Up Obesity Medicine Visit Mini-Clinical Evaluation Exercises (Mini-CEXs)

*Note:* The group visit Mini-CEX is available upon request from the corresponding author.

**Supplemental Materials 1: Baseline Faculty Evaluation of Obesity Medicine Fellow, Program Evaluation by the Fellow and Faculty Evaluation of the Program**

Baseline Faculty Evaluation of Obesity Medicine Fellow

Instructions:

*This evaluation should be a baseline assessment based on your observations of the obesity medicine fellow. Typical fellows are expected to begin at a Level 1-2 of competency at this stage of their health professional careers. Occasionally, they may be above or below this level. Please also provide specific positive observations and suggestions for improvement.*

1* Please estimate the number of hours that you have worked with this fellow over the last month:

Range from 1 to 1000

PATIENT CARE AND PROCEDURAL SKILLS COMPETENCIES


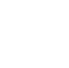

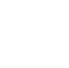
2* PATIENT CARE AND PROCEDURAL SKILLS 1. Elicits comprehensive obesity-focused medical history.

| **Level 1** | **Level 2** | **Level 3** 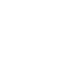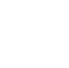 | **Level 4** | **Level 5** |
| --- | --- | --- | --- | --- |
| Complete history taking | Complete history taking | Complete history taking | Complete history taking | Complete history taking |
| is insensitive, | is reasonably sensitive | is patient and family- | is patient and family- | is patient and family- |
| disorganized, and/ or | and uses people-first | centered, uses people- | centered, uses people- | centered, uses |
| misses important details | language, is fairly | first language, is | first language, is | peoplefirst language, is |
| for patients with simple | organized and complete, | organized and complete, | organized and complete, | organized and complete, |
| weight management | missing few important | is appropriate for | is appropriate for | is appropriate for |
| challenges. | details for patients with | gathering obesity-related | gathering obesity-related | gathering obesity-related |
|  | simple weight | information, and is | information, and is | information, and is |
|  | management challenges. | efficient for patients with | efficient for patients with | efficient for patients with |
|  |  | SIMPLE weight | MODERATE weight | COMPLEX clinical and |
|  |  | management challenges. | management challenges | psychological weight |
|  |  |  |  | management challenges. |
| 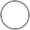 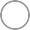 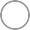 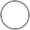 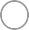 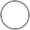 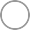 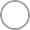 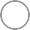 | | | | |


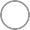
 Unable to evaluate Comment

3* PATIENT CARE AND PROCEDURAL SKILLS 2. Performs and documents a comprehensive physical examination for the assessment of obesity.

| **Level 1** | **Level 2** | **Level 3** | **Level 4** | **Level 5** |
| --- | --- | --- | --- | --- |
| Physical examination is | Physical examination | Physical examination is | Physical examination is | Physical examination is |
| incomplete, techniques | contains key | usually complete and | consistently complete, | consistently complete, |
| are inaccurate and | components; techniques | focused, technique is | systematic, and focused | systematic, and focused |
| insensitive to patient’s | are fairly appropriate and | mostly accurate, usually | appropriately using | appropriately using |
| modesty and comfort | fairly sensitive to | ensures patient’s | accurate techniques that | accurate techniques that |
| during physical | patient’s modesty and | modesty and comfort | ensure patient’s | ensure patient’s |
| examination; incomplete | comfort during physical | during physical | modesty and comfort; | modesty and comfort; |
| documentation of | examination; fairly | examination; | documentation of | documentation of |
| findings. | complete documentation | documentation of | findings is complete and | findings is complete and |
|  | of findings. | findings are mostly | well organized for | well organized for |
|  |  | complete and organized | patients with | patients with COMPLEX |
|  |  | for patients with SIMPLE | MODERATE weight | weight management |
|  |  | weight management | management challenges. | challenges. |
|  |  | challenges. |  |  |
| 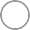 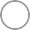 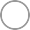 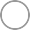 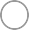 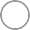 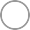 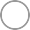 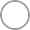 | | | | |


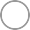
 Unable to evaluate Comment

4* PATIENT CARE AND PROCEDURAL SKILLS 3. Effectively applies clinical reasoning skills when ordering and interpreting appropriate laboratory and diagnostic tests during the evaluation of patients with obesity.

| **Level 1** | **Level 2** | **Level 3** | **Level 4** | **Level 5** |
| --- | --- | --- | --- | --- |
| Use of evidencebased | Use of laboratory and | Use of laboratory and | Use of laboratory and | Use of laboratory and |
| laboratory and | diagnostic tests is | diagnostic tests is | diagnostic tests is | diagnostic tests is |
| diagnostics tests is | organized, clinical | organized, clinical | organized and efficient | organized and efficient |
| incomplete or | reasoning and | reasoning and | without extraneous | without extraneous |
| disorganized, orders | interpretation are | interpretation of data | diagnostics for | diagnostics in |
| unnecessary or non- | missing a few key | support differential | MODERATELY | COMPLEX cases of |
| evidencebased tests, | components but | diagnosis and include | challenging cases of | obesity, clinical |
| clinical reasoning and | differential diagnosis is | the diagnosis for | obesity, clinical | reasoning and |
| interpretation of data is | supported. | SIMPLE cases of | reasoning and | interpretation of data are |
| limited, and differential |  | obesity. | interpretation of data are | accurate and support |
| diagnosis is limited or |  |  | accurate and support | the correct diagnosis |
| not supported. |  |  | the correct diagnosis. |  |
| 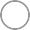 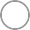 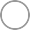 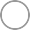 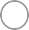 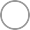 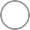 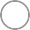 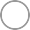 | | | | |


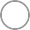
 Unable to evaluate Comment

5* PATIENT CARE AND PROCEDURAL SKILLS 4. Utilizes evidence-based models of health behavior change to assess patients’ readiness to change in order to effectively counsel patients for weight management.

| **Level 1** | **Level 2** | **Level 3** | **Level 4** | **Level 5** |
| --- | --- | --- | --- | --- |
| Counseling for weight | Counseling for weight | Counseling for weight | Counseling for weight | Counseling for weight |
| management is | management is | management is usually | management is | management is |
| performed, but | sometimes performed | performed using | consistently performed | consistently performed |
| evidence-based models | using evidence-based | evidence-based models | using evidence-based | using evidence-based |
| of health behavior | models of health | of health behavior | models of health | models of health |
| change are not used. | behavior change. Goals | change. Goals provided | behavior change. Goals | behavior change. Goals |
| The goals are | provided are sometimes | are clear, thorough, and | provided are clear, | provided are clear, |
| incomplete and | clear, thorough, and | patientcentered. | thorough, and | thorough, and |
| provider-centered. | patient-centered for | Counseling is usually | patientcentered. | patientcentered. |
|  | patients with simple | efficient for patients with | Counseling is | Counseling is |
|  | weight management | SIMPLE weight | consistently efficient for | consistently efficient for |
|  | challenges. | management challenges. | patients with | patients with COMPLEX |
|  |  |  | MODERATE weight | weight management |
|  |  |  | management challenges. | challenges. |
| 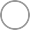 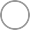 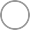 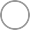 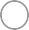 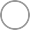 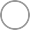 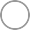 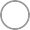 | | | | |


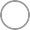
 Unable to evaluate Comment

6* PATIENT CARE AND PROCEDURAL SKILLS 5. Engages the patients and their support systems in shared decision-making by incorporating their values and preferences in the development of a comprehensive personalized obesity management care plan.

| **Level 1** | **Level 2** | **Level 3** | **Level 4** | **Level 5** |
| --- | --- | --- | --- | --- |
| Patients and their | Patients and their | Patients and their | Patients and their | Patients and their |
| support systems are | support systems are | support systems are | support systems are | support systems are |
| rarely engaged in shared | SOMETIMES engaged in | USUALLY engaged in | CONSISTENTLY | consistently engaged in |
| decisionmaking, and the | shared decision-making | shared decision-making | engaged in shared | shared decision-making |
| management plan is | to develop a FAIRLY | to develop a | decision-making to | to develop a |
| nonpersonalized for | personalized obesity | COMPREHENSIVE | develop a | comprehensive |
| patients with simple | management plan for | personalized obesity | comprehensive | personalized obesity |
| weight management | patients with SIMPLE | management plan for | personalized obesity | management plan for |
| challenges. | weight management | patients with SIMPLE | management plan for | patients with COMPLEX |
|  | challenges. | weight management | patients with | weight management |
|  |  | challenges. | MODERATE weight | challenges. |
|  |  |  | management challenges. |  |
| 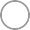 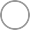 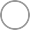 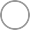 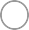 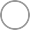 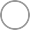 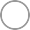 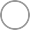 | | | | |


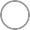
 Unable to evaluate Comment

MEDICAL KNOWLEDGE COMPETENCIES

7* MEDICAL KNOWLEDGE 1. Demonstrates knowledge of obesity epidemiology.

| **Level 1** | **Level 2** | **Level 3** | **Level 4** | **Level 5** |
| --- | --- | --- | --- | --- |
| Lacks basic knowledge | Has BASIC knowledge | Has AVERAGE | Has ABOVE AVERAGE | Has EXCEPTIONAL |
| of overweight and | of overweight and | knowledge of | knowledge of | knowledge of |
| obesity incidence and | obesity incidence and | overweight and obesity | overweight and obesity | overweight and obesity |
| prevalence, effects on | prevalence, effects on | incidence, prevalence, | incidence, prevalence, | incidence, prevalence, |
| morbidity and mortality, | morbidity and mortality, | and trends, effects on | and trends, effects on | and trends, effects on |
| and demographic | and demographic | morbidity and mortality, | morbidity and mortality, | morbidity and mortality, |
| associations and | associations and | and demographic | and demographic | and demographic |
| distributions for children | distributions for children | associations and | associations and | associations and |
| and adults. Cannot | and adults. Can identify | distributions for children | distributions for children | distributions for children |
| identify common | common environmental, | and adults. | and adults. | and adults. |
| environmental, | socioeconomic, and | Demonstrates | Demonstrates | Demonstrates |
| socioeconomic, and | behavioral contributors | knowledge of common | knowledge of common | knowledge of common, |
| behavioral contributors | to the obesity epidemic | environmental, | and subtle | subtle, and theorized |
| to the obesity epidemic | at the population level. | socioeconomic, and | environmental, | environmental, |
| at the population level. |  | behavioral contributors | socioeconomic, and | socioeconomic, and |
|  |  | to the obesity epidemic | behavioral contributors | behavioral contributors |
|  |  | at the population level | to the obesity epidemic | to the obesity epidemic |
|  |  |  | at the population level. | at the population level. |
| 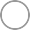 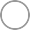 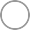 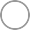 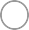 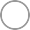 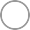 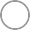 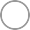 | | | | |


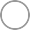
Unable to evaluate

Comment

8* MEDICAL KNOWLEDGE 2. Demonstrates knowledge of energy homeostasis and weight regulation.

| **Level 1** | **Level 2** | **Level 3** | **Level 4** | **Level 5** |
| --- | --- | --- | --- | --- |
| Lacks basic knowledge | Has BASIC knowledge | Has AVERAGE | Has ABOVE AVERAGE | Has EXCEPTIONAL |
| of energy homeostasis | of energy homeostasis | knowledge of energy | knowledge of energy | knowledge of energy |
| and weight regulation, | and weight regulation, | homeostasis and weight | homeostasis and weight | homeostasis and weight |
| including cellular and | including cellular and | regulation, and can | regulation, including | regulation, including |
| biochemical energy | biochemical energy | apply that knowledge to | entero-neuroendocrine | entero-neuroendocrine |
| storage/ transfer, | storage/ transfer, | the clinical care of | physiology, and can | physiology, and can |
| thermodynamics, and | thermodynamics, and | patients. | apply that knowledge to | apply that knowledge to |
| energy expenditure. | energy expenditure. |  | the clinical care of | the clinical care of |
|  |  |  | patients. | complex patients |
| 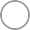 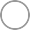 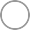 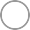 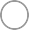 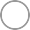 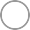 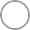 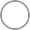 | | | | |


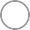
Unable to evaluate

Comment

9* MEDICAL KNOWLEDGE 3. Demonstrates knowledge of anthropometric measurements and clinical assessments of energy expenditure. Anthropometric or body composition measurements may include weight for length, BMI, BMI percentile, BMI z-score, BMI % relative to 95th percentile, waist circumference (WC), and waist-to-hip ratio (WHR).

| **Level 1** | **Level 2** | **Level 3** | **Level 4** | **Level 5** |
| --- | --- | --- | --- | --- |
| Lacks basic knowledge | Has BASIC knowledge | Has AVERAGE | Has ABOVE AVERAGE | Has EXCEPTIONAL |
| of body composition | of body composition | knowledge of body | knowledge of body | knowledge of body |
| measurements and | measurements and | composition | composition | composition |
| clinical assessments of | clinical assessments of | measurements (including | measurements (including | measurements (including |
| energy expenditure (e.g., | energy expenditure (e.g., | bioimpedance, skinfold | bioimpedance, skinfold | bioimpedance, skinfold |
| Harris-Benedict (HB) | HB and MSJ equations). | measurements) and | measurements, DXA) | measurements, DXA, |
| and Miﬄin-St. Jeor |  | clinical assessments of | and clinical assessments | cross-sectional imaging, |
| (MSJ) equations). |  | energy expenditure (e.g., | of energy expenditure | underwater weighing) |
|  |  | HB and MSJ equations), | (e.g., HB and MSJ | and clinical assessments |
|  |  | and can apply that | equations, indirect | of energy expenditure |
|  |  | knowledge to the clinical | calorimetry), and can | (e.g., HB and MSJ |
|  |  | care of patients. | apply that knowledge to | equations, indirect |
|  |  |  | the clinical care of | calorimetry, doubly- |
|  |  |  | patients. Recognizes | labeled water, metabolic |
|  |  |  | indications, limitations, | chamber), and can apply |
|  |  |  | and utility of various | that knowledge to the |
|  |  |  | measurements. | clinical care of complex |
|  |  |  |  | patients. Can distinguish |
|  |  |  |  | nuanced differences |
|  |  |  |  | between various |
|  |  |  |  | technologies and |
|  |  |  |  | measurements, and is |
|  |  |  |  | able to apply the |
|  |  |  |  | appropriate study for |
|  |  |  |  | clinical or investigational |
|  |  |  |  | purposes. |
| 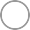 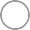 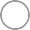 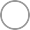 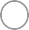 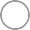 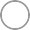 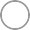 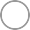 | | | | |


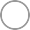
 Unable to evaluate Comment

10* MEDICAL KNOWLEDGE 4. Demonstrates knowledge of the etiologies, mechanisms and biology of obesity.

| **Level 1** | **Level 2** | **Level 3** | **Level 4** | **Level 5** |
| --- | --- | --- | --- | --- |
| Lacks basic knowledge | Has BASIC knowledge | Has AVERAGE | Has ABOVE AVERAGE | Has COMPREHENSIVE |
| of the etiologies, | of the etiologies, | knowledge of the | knowledge of the | knowledge of the |
| mechanisms, and | mechanisms, and | etiologies, mechanisms, | etiologies, mechanisms, | etiologies, mechanisms, |
| biology of obesity. | biology of obesity. | and biology of obesity, | and biology of obesity, | and biology of obesity, |
|  |  | and can apply that | and can apply that | and can apply that |
|  |  | knowledge to the clinical | knowledge to the clinical | knowledge to the clinical |
|  |  | care of patients. | care of patients. | care of complex |
|  |  |  |  | patients. |
| 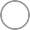 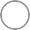 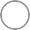 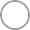 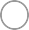 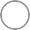 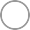 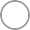 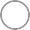 | | | | |


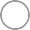
 Unable to evaluate Comment

11* MEDICAL KNOWLEDGE 5. Demonstrates knowledge of obesity-related comorbidities and the corresponding benefits of body mass index (BMI) reduction.

| **Level 1** | **Level 2** | **Level 3** | **Level 4** | **Level 5** |
| --- | --- | --- | --- | --- |
| Lacks basic knowledge | Has BASIC knowledge | Has AVERAGE | Has ABOVE AVERAGE | Has EXCEPTIONAL |
| of obesity-related | of obesity-related | knowledge of obesity- | knowledge of obesity- | knowledge of obesity- |
| comorbidities and the | comorbidities and the | related comorbidities | related comorbidities | related comorbidities |
| corresponding benefits | corresponding benefits | and the corresponding | and the corresponding | and the corresponding |
| of BMI reduction. | of BMI reduction. | benefits of BMI | benefits of BMI | benefits of BMI |
|  |  | reduction, and can apply | reduction, and can apply | reduction, and can apply |
|  |  | that knowledge to the | that knowledge to the | that knowledge to the |
|  |  | clinical care of patients. | clinical care of patients. | clinical care of complex |
|  |  |  |  | patients |
| 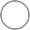 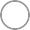 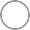 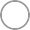 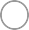 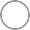 | | | | |

Unable to evaluate Comment

12* MEDICAL KNOWLEDGE 6. Applies knowledge of the principles of primary, secondary, and tertiary prevention of obesity to the development of a comprehensive, personalized obesity management care plan. Please consider the following definitions in the context of obesity: Primary prevention=prevent development of overweight/obesity; Secondary prevention=reduce BMI to prevent development of weight-related complications; Tertiary prevention=reduce BMI to prevent progression or worsening of established weight-related complications.

| **Level 1** | **Level 2** | **Level 3** | **Level 4** | **Level 5** |
| --- | --- | --- | --- | --- |
| Lacks basic knowledge | Has BASIC knowledge | Has AVERAGE | Has ABOVE AVERAGE | Has EXCEPTIONAL |
| of the principles of | of the principles of | knowledge of the | knowledge of the | knowledge of the |
| primary, secondary, and | primary, secondary, and | principles of primary, | principles of primary, | principles of primary, |
| tertiary prevention for | tertiary prevention for | secondary, and tertiary | secondary, and tertiary | secondary, and tertiary |
| the prevention and | the prevention and | prevention for the | prevention for the | prevention for the |
| treatment of obesity. | treatment of obesity. | prevention and | prevention and | prevention and |
|  |  | treatment of obesity, and | treatment of obesity, and | treatment of obesity, and |
|  |  | can apply that | can apply that | can apply that |
|  |  | knowledge to the clinical | knowledge to the clinical | knowledge to the clinical |
|  |  | care of patients. | care of patients. | care of complex |
|  |  |  |  | patients. |
|  | | | | |

Unable to evaluate Comment

13* MEDICAL KNOWLEDGE 7. Applies knowledge of obesity treatment guidelines to the development of a comprehensive, personalized obesity management care plan.

| **Level 1** | **Level 2** | **Level 3** | **Level 4** | **Level 5** |
| --- | --- | --- | --- | --- |
| Lacks basic knowledge | Has BASIC knowledge | Has AVERAGE | Has ABOVE AVERAGE | Has EXCEPTIONAL |
| of guidelines for the | of guidelines for the | knowledge of guidelines | knowledge of guidelines | knowledge of guidelines |
| treatment of obesity. | treatment of obesity. | for the treatment of | for the treatment of | for the treatment of |
|  |  | obesity, and can apply | obesity, and can apply | obesity, and can apply |
|  |  | that knowledge to the | that knowledge to the | that knowledge to the |
|  |  | clinical care of patients. | clinical care of patients. | clinical care of complex |
|  |  |  | Recognizes limitations of | patients. Recognizes the |
|  |  |  | guidelines with respect | evidence base for |
|  |  |  | to individual patient | obesity treatment |
|  |  |  | care. | guidelines, limitations of |
|  |  |  |  | guidelines with respect |
|  |  |  |  | to individual patient |
|  |  |  |  | care, and areas of |
|  |  |  |  | continued scientific |
|  |  |  |  | uncertainty. |
|  | | | | |

Unable to evaluate Comment

14* MEDICAL KNOWLEDGE 8. Applies knowledge of using nutrition interventions to develop a comprehensive, personalized obesity management care plan.

| **Level 1** | **Level 2** | **Level 3** | **Level 4** | **Level 5** |
| --- | --- | --- | --- | --- |
| Lacks basic knowledge | Has BASIC knowledge | Has AVERAGE | Has ABOVE AVERAGE | Has EXCEPTIONAL |
| of nutrition interventions | of nutrition interventions | knowledge of nutrition | knowledge of nutrition | knowledge of nutrition |
| for the treatment of | for the treatment of | interventions for the | interventions for the | interventions for the |
| obesity. | obesity. | treatment of obesity, and | treatment of obesity, and | treatment of obesity, and |
|  |  | can apply that | can apply that | can apply that |
|  |  | knowledge to the clinical | knowledge to the clinical | knowledge to the clinical |
|  |  | care of patients. | care of patients. | care of complex |
|  |  |  |  | patients. |
|  | | | | |

Unable to evaluate Comment

15* MEDICAL KNOWLEDGE 9. Applies knowledge of using physical activity interventions to develop a comprehensive, personalized obesity management care plan.

| **Level 1** | **Level 2** | **Level 3** | **Level 4** | **Level 5** |
| --- | --- | --- | --- | --- |
| Lacks basic knowledge | Has BASIC knowledge | Has AVERAGE | Has ABOVE AVERAGE | Has EXCEPTIONAL |
| of physical activity | of physical activity | knowledge of physical | knowledge of physical | knowledge of physical |
| guidelines and | interventions for the | activity interventions for | activity interventions for | activity interventions for |
| interventions for the | treatment of obesity. | the treatment of obesity, | the treatment of obesity, | the treatment of obesity, |
| treatment of obesity. |  | and can apply that | and can apply that | and can apply that |
|  |  | knowledge to the clinical | knowledge to the clinical | knowledge to the clinical |
|  |  | care of patients. | care of patients. | care of complex |
|  |  |  |  | patients. |
|  | | | | |

Unable to evaluate Comment

16* MEDICAL KNOWLEDGE 10. Applies knowledge of using behavioral interventions to develop a comprehensive, personalized obesity management care plan. Behavioral interventions may include behavior therapy strategies, psychological counseling, sleep regulation, stress reduction.

| **Level 1** | **Level 2** | **Level 3** | **Level 4** | **Level 5** |
| --- | --- | --- | --- | --- |
| Lacks basic knowledge | Has BASIC knowledge | Has AVERAGE | Has ABOVE AVERAGE | Has EXCEPTIONAL |
| of behavioral | of behavioral | knowledge of behavioral | knowledge of behavioral | knowledge of behavioral |
| interventions for the | interventions for the | interventions for the | interventions for the | interventions for the |
| treatment of obesity. | treatment of obesity. | treatment of obesity, and | treatment of obesity, and | treatment of obesity, and |
|  |  | can apply that | can apply that | can apply that |
|  |  | knowledge to the clinical | knowledge to the clinical | knowledge to the clinical |
|  |  | care of patients. | care of patients. | care of complex |
|  |  |  |  | patients. |
|  | | | | |

Unable to evaluate Comment

17* MEDICAL KNOWLEDGE 11. Applies knowledge of using pharmacological treatments of obesity as part of a comprehensive, personalized obesity management care plan.

| **Level 1** | **Level 2** | **Level 3** | **Level 4** | **Level 5** |
| --- | --- | --- | --- | --- |
| Does not recognize | Recognizes anti-obesity | Has AVERAGE | Has ABOVE AVERAGE | Has EXCEPTIONAL |
| antiobesity medication | medication as an | knowledge of the age- | knowledge of the age- | knowledge of the age- |
| as an appropriate form | appropriate form of | appropriate | appropriate | appropriate |
| of therapy. Lacks basic | therapy, and has BASIC | pharmacotherapeutic | pharmacotherapeutic | pharmacotherapeutic |
| knowledge of the age- | knowledge of the age- | options for the treatment | options for the treatment | options for the treatment |
| appropriate | appropriate | of obesity, including their | of obesity, including their | of obesity, including their |
| pharmacotherapeutic | pharmacotherapeutic | indications, | indications, | indications, |
| options for the treatment | options for the treatment | contraindications, side | contraindications, side | contraindications, side |
| of obesity, including | of obesity, including their | effects, and mechanisms | effects, and mechanisms | effects, and mechanisms |
| their indications, | indications, | of action, and can apply | of action, and can apply | of action, and can apply |
| contraindications, side | contraindications, side | that knowledge to the | that knowledge to the | that knowledge to the |
| effects, and | effects, and mechanisms | clinical care of patients. | clinical care of patients. | clinical care of complex |
| mechanisms of action. | of action. |  |  | patients. |
|  | | | | |

Unable to evaluate Comment

18* MEDICAL KNOWLEDGE 12. Applies knowledge of the surgical treatments of obesity as part of a comprehensive, personalized obesity management care plan.

| **Level 1** | **Level 2** | **Level 3** | **Level 4** | **Level 5** |
| --- | --- | --- | --- | --- |
| Does not recognize | Recognizes age- | Has AVERAGE | Has ABOVE AVERAGE | Has EXCEPTIONAL |
| bariatric surgery as an | appropriate bariatric | knowledge of the | knowledge of the | knowledge of the |
| appropriate form of | surgery as an | surgical options for the | surgical options for the | evidence-based patient |
| therapy or the options | appropriate form of | treatment of obesity, | treatment of obesity, | selection for surgical |
| available. Lacks basic | therapy and the options | mechanisms of action, | mechanisms of action, | options for the treatment |
| knowledge of the | available. Has BASIC | and metabolic/clinical | and metabolic/ clinical | of obesity, mechanisms |
| mechanisms of action | knowledge of the | outcomes, and can | outcomes, and can | of action, and |
| and metabolic/clinical | mechanisms of action | apply that knowledge to | apply that knowledge to | metabolic/clinical |
| outcomes. | and metabolic/clinical | the clinical care of | the pre- and | outcomes, and can |
|  | outcomes. | patients. | postoperative clinical | apply that knowledge to |
|  |  |  | care of patients. | the pre- and |
|  |  |  |  | postoperative clinical |
|  |  |  |  | care of complex |
|  |  |  |  | patients. |
|  | | | | |

Unable to evaluate Comment

19* MEDICAL KNOWLEDGE 13. Applies knowledge of emerging treatment modalities for obesity to the development of a comprehensive, personalized obesity management care plan. Emerging treatment modalities may include devices, medications, procedures/surgeries, endoscopic bariatric therapies (EBTs), electronic applications/technologies.

| **Level 1** | **Level 2** | **Level 3** | **Level 4** | **Level 5** |
| --- | --- | --- | --- | --- |
| Lacks basic knowledge | Has BASIC knowledge | Has AVERAGE | Has ABOVE AVERAGE | Has EXCEPTIONAL |
| of emerging modalities | of emerging modalities | knowledge of emerging | knowledge of emerging | knowledge of emerging |
| for the treatment of | for the treatment of | modalities for the | modalities for the | modalities for the |
| obesity. | obesity. | treatment of obesity, and | treatment of obesity, and | treatment of obesity, and |
|  |  | can apply that | can apply that | can apply that |
|  |  | knowledge to the clinical | knowledge to the clinical | knowledge to the clinical |
|  |  | care of patients. | care of patients. | care of complex |
|  |  |  |  | patients. |
|  | | | | |

Unable to evaluate Comment

PRACTICE-BASED LEARNING AND IMPROVEMENT COMPETENCIES

20* PRACTICE-BASED LEARNING AND IMPROVEMENT 1. Evaluates strengths and deficiencies in knowledge of obesity medicine and sets and achieves goals for improvement.

| **Level 1** | **Level 2** | **Level 3** | **Level 4** | **Level 5** |
| --- | --- | --- | --- | --- |
| Unable to evaluate | Able to evaluate FEW | Able to evaluate SOME | Able to evaluate MOST | Able to |
| strengths and | strengths and | strengths and | strengths and | COMPREHENSIVELY |
| deficiencies in | deficiencies in | deficiencies in | deficiencies in | evaluate strengths and |
| knowledge of obesity | knowledge of obesity | knowledge of obesity | knowledge of obesity | deficiencies in |
| medicine, and unable to | medicine, and able to | medicine, and able to | medicine, and able to | knowledge of obesity |
| set goals for | set and achieve limited | set and achieve some | set and achieve most | medicine, and able to |
| improvement. | goals for improvement. | goals for improvement. | goals for improvement. | consistently set and |
|  |  |  |  | achieve goals for |
|  |  |  |  | improvement. |
|  | | | | |

Unable to evaluate Comment

21* PRACTICE-BASED LEARNING AND IMPROVEMENT 2. Analyzes practice systems using quality improvement methods to monitor and optimize obesity care.

| **Level 1** | **Level 2** | **Level 3** | **Level 4** | **Level 5** |
| --- | --- | --- | --- | --- |
| Unable to analyze | Able to analyze SOME | Able to analyze a WIDE | Able to analyze more | Consistently able to |
| practice systems using | practice systems using | RANGE of BASIC | ADVANCED practice | analyze COMPLEX |
| quality improvement | quality improvement | practice systems using | systems using quality | practice systems using |
| methods to monitor and | methods to monitor and | quality improvement | improvement methods | quality improvement |
| optimize obesity care. | optimize obesity care. | methods to monitor and | to monitor and optimize | methods to monitor and |
|  |  | optimize obesity care. | obesity care. | optimize obesity care. |
|  | | | | |

Unable to evaluate Comment

22* PRACTICE-BASED LEARNING AND IMPROVEMENT 3. Utilizes resources to locate, interpret, and apply evidence from scientific studies regarding obesity treatment and its co-morbidities.

| **Level 1** | **Level 2** | **Level 3** | **Level 4** | **Level 5** |
| --- | --- | --- | --- | --- |
| Unable to utilize | Able to utilize resources | Able to utilize resources | Able to utilize resources | CONSISTENTLY utilizes |
| resources to locate, | to locate evidence, but | to locate evidence and | to locate and interpret | resources to locate, |
| interpret, or apply | unable to interpret or | BEGINNING to interpret, | evidence, and BEGINS | interpret, and apply |
| evidence from scientific | apply evidence from | but NOT able to apply | to apply evidence from | evidence from scientific |
| studies regarding | scientific studies | evidence from scientific | scientific studies | studies regarding |
| obesity treatment and its | regarding obesity | studies regarding | regarding obesity | obesity treatment and its |
| co-morbidities. | treatment and its co- | obesity treatment and its | treatment and its co- | co-morbidities. |
|  | morbidities. | co-morbidities. | morbidities. |  |
|  | | | | |

Unable to evaluate Comment

23* PRACTICE-BASED LEARNING AND IMPROVEMENT 4. Uses information technology related to obesity treatment to optimize delivery of care including EHRs, software applications, and related devices (i.e., accelerometers, resting metabolic rate, and body composition analysis technology).

| **Level 1** | **Level 2** | **Level 3** | **Level 4** | **Level 5** |
| --- | --- | --- | --- | --- |
| Unable to use any forms | Able to use a FEW | Able to use BASIC forms | Able to use MOST forms | VERY PROFICIENT in |
| of information | LIMITED forms of | of information | of information | the use of information |
| technology related to | information technology | technology related to | technology related to | technology related to |
| obesity treatment to | related to obesity | obesity treatment to | obesity treatment to | obesity treatment to |
| optimize delivery of care | treatment, but with an | optimize delivery of care | optimize delivery of care | optimize delivery of care |
| including EHRs, | incomplete | including EHRs, | including EHRs, | including EHRs, |
| software applications, | comprehension, and | software applications, | software applications, | software applications, |
| and related devices. | therefore UNABLE to | and related devices. | and related devices. | and related devices. |
|  | optimize delivery of care |  |  |  |
|  | including EHRs, |  |  |  |
|  | software applications, |  |  |  |
|  | and related devices. |  |  |  |
|  | | | | |

Unable to evaluate Comment

24* PRACTICE-BASED LEARNING AND IMPROVEMENT 5. Effectively educates patients, students, residents, and other health professionals on the disease of obesity

| **Level 1** | **Level 2** | **Level 3** | **Level 4** | **Level 5** |
| --- | --- | --- | --- | --- |
| Unable to educate | Provides INEFFECTIVE | Effectively provides | Effectively educates | Consistently and |
| patients, students, | or incomplete education | BASIC education to | patients, students, | effectively educates |
| residents, and other | to patients, students, | patients, students, | residents, and other | patients, students, |
| health professionals on | residents, and other | residents, and other | health professionals on | residents, and other |
| the disease of obesity. | health professionals on | health professionals on | the disease of obesity in | health professionals on |
|  | the disease of obesity. | the disease of obesity in | common, more | the disease of obesity in |
|  |  | basic clinical cases. | ADVANCED clinical | a full spectrum of |
|  |  |  | cases. | scenarios, including |
|  |  |  |  | CHALLENGING clinical |
|  |  |  |  | cases. |
|  | | | | |

Unable to evaluate Comment

INTERPERSONAL AND COMMUNICATION SKILLS COMPETENCIES

25* INTERPERSONAL AND COMMUNICATION SKILLS 1. Uses appropriate language in verbal, nonverbal, and written communication that is non-biased, non-judgmental, respectful, and empathetic when communicating with patients with obesity. Appropriate verbal communication includes people-first and weight-friendly language.

| **Level 1** | **Level 2** | **Level 3** | **Level 4** | **Level 5** |
| --- | --- | --- | --- | --- |
| Verbal, nonverbal, and | OCCASIONALLY utilizes | Utilizes verbal, | CONSISTENTLY utilizes | CONSISTENTLY and |
| written communication | verbal, nonverbal, and | nonverbal, and written | appropriate verbal, | EFFORTLESSLY utilizes |
| is biased, judgmental, | written communication | communication that is | nonverbal, and written | appropriate verbal, |
| disrespectful, and/ or | that is inappropriate | appropriate when | communication that is | nonverbal, and written |
| not empathetic when | when engaging with | engaging with PATIENTS | tailored to individual | communication that is |
| communicating with | PATIENTS WITH | WITH OBESITY. | circumstances when | clear, concise, and |
| patients with obesity. | OBESITY, but corrects |  | engaging with PATIENTS | tailored to individual |
|  | when pointed out. |  | WITH OBESITY, | circumstances when |
|  |  |  | including challenging | engaging with PATIENTS |
|  |  |  | situations. | WITH OBESITY in all |
|  |  |  |  | situations. |
|  | | | | |

Unable to evaluate Comment

26* INTERPERSONAL AND COMMUNICATION SKILLS 2. Uses appropriate language in verbal, nonverbal, and written communication that is non-biased, non-judgmental, respectful, and empathetic when communicating about patients with obesity with colleagues within one’s profession and other members of the healthcare team. Appropriate verbal communication includes people-first and weight-friendly language.

| **Level 1** | **Level 2** | **Level 3** | **Level 4** | **Level 5** |
| --- | --- | --- | --- | --- |
| Verbal, nonverbal, and | OCCASIONALLY verbal, | Utilizes verbal, | CONSISTENTLY utilizes | CONSISTENTLY and |
| written communication | nonverbal, and written | nonverbal, and written | appropriate verbal, | EFFORTLESSLY utilizes |
| is biased, judgmental, | communication that is | communication that is | nonverbal, and written | appropriate verbal, |
| and/or disrespectful | inappropriate when | appropriate when | communication that is | nonverbal, and written |
| when communicating | engaging HEALTHCARE | engaging HEALTHCARE | tailored to individual | communication that is |
| with healthcare | PROFESSIONALS in | PROFESSIONALS in | circumstances when | clear, concise, and |
| professionals in clinical | clinical and non-clinical | clinical and non-clinical | engaging HEALTHCARE | tailored to individual |
| and non-clinical settings | settings, but corrects | settings. | PROFESSIONALS in | circumstances when |
| (non-clinical settings | when pointed out. |  | clinical and non-clinical | engaging HEALTHCARE |
| includes discussion |  |  | settings, including | PROFESSIONALS in |
| outside of patient care |  |  | challenging situations. | clinical and non-clinical |
| settings such as back |  |  |  | settings and in all |
| office, hallways, |  |  |  | situations. |
| cafeteria, or social |  |  |  |  |
| settings). |  |  |  |  |
|  | | | | |

Unable to evaluate Comment

27* INTERPERSONAL AND COMMUNICATION SKILLS 3. Demonstrates awareness of different cultural views regarding perceptions of desired weight and preferred body shape when communicating with the patient, family, and other members of the healthcare team. Diversity and preferences includes language, ideal body weight and shape, family rituals, lifestyle practices, food choices, and/or use of alternative medicines.

| **Level 1** | **Level 2** | **Level 3** | **Level 4** | **Level 5** |
| --- | --- | --- | --- | --- |
| Exhibits specific | Exhibits LACK of | Demonstrates an | CONSISTENTLY | Consistently |
| episodes of cultural | APPRECIATION for | appreciation of cultural | demonstrates an | demonstrates an |
| insensitivity when | cultural diversity and | diversity and | appreciation of cultural | appreciation of cultural |
| communicating with | preferences when | preferences when | diversity and | diversity and |
| others (others includes | communicating with | communicating with | preferences when | preferences when |
| the patient, family, and | others, but corrects | others and makes use of | communicating with | communicating with |
| other members of the | when pointed out. | interpreter services | others, consistently uses | others in ALL situations, |
| healthcare team). |  | when indicated. | interpreter services | role models and teaches |
|  |  |  | when indicated and in | these qualities to other |
|  |  |  | challenging situations, | members of the |
|  |  |  | addresses adversity or | healthcare team. |
|  |  |  | denial to change. | Recognizes and |
|  |  |  | Thoroughly explores | addresses implicit and |
|  |  |  | cultural barriers. | explicit bias in patients, |
|  |  |  | Recognizes implicit and | family, staff, and self. |
|  |  |  | explicit bias in patients, |  |
|  |  |  | family, staff, and self. |  |
|  | | | | |

Unable to evaluate Comment

PROFESSIONALISM COMPETENCIES

28* PROFESSIONALISM 1. Demonstrates ethical behavior and integrity when counseling patients and their families who are living with overweight or obesity.

| **Level 1** | **Level 2** | **Level 3** | **Level 4** | **Level 5** |
| --- | --- | --- | --- | --- |
| Exhibits LACK of | Exhibits LACK of | Exhibits competence, | CONSISTENTLY exhibits | Consistently exhibits |
| competence, honesty, | competence, honesty, | honesty, responsibility, | competence, honesty, | competence, honesty, |
| responsibility, and/ or | responsibility, | trustworthiness, and | responsibility, | responsibility, |
| trustworthiness and | trustworthiness, and/or | lack of bias when | trustworthiness, and | trustworthiness, and |
| exhibits bias when | exhibits bias when | counseling most if not all | lack of bias when | lack of bias when |
| counseling patients and | counseling patients and | patients and families | counseling patients and | counseling patients and |
| families who are living | families who are living | who are living with | families who are living | families who are living |
| with overweight or | with overweight or | overweight or obesity. | with overweight or | with overweight or |
| obesity, and FAILS to | obesity, but CORRECTS |  | obesity, including in | obesity in ALL situations, |
| acknowledge or correct | when pointed out. |  | challenging situations. | and acts as a ROLE |
| when pointed out. |  |  |  | MODEL to teach these |
|  |  |  |  | qualities to others. |
|  | | | | |

Unable to evaluate Comment

29* PROFESSIONALISM 2. Displays compassion and respect toward all patients and families who are living with overweight or obesity.

| **Level 1** | **Level 2** | **Level 3** | **Level 4** | **Level 5** |
| --- | --- | --- | --- | --- |
| Exhibits lack of | Exhibits lack of | Exhibits compassionate | CONSISTENTLY exhibits | Consistently exhibits |
| compassionate, | compassionate, | and respectful behavior | compassionate and | compassionate and |
| respectful behavior | respectful behavior and | and lack of bias when | respectful behavior and | respectful behavior and |
| and/or exhibits bias | exhibits bias when | working with most if not | lack of bias when | lack of bias when |
| when working with | working with patients | all patients and families | working with patients | working with patients |
| patients and families | and families who are | who are living with | and families who are | and families who are |
| who are living with | living with overweight or | overweight or obesity. | living with overweight or | living with overweight or |
| overweight or obesity, | obesity, but CORRECTS |  | obesity, including in | obesity in ALL situations, |
| and FAILS to | when pointed out. |  | challenging situations. | and acts as a ROLE |
| acknowledge or correct |  |  |  | MODEL to teach these |
| when pointed out. |  |  |  | qualities to others. |
|  | | | | |

Unable to evaluate Comment

SYSTEMS-BASED PRACTICE COMPETENCIES

30* SYSTEMS-BASED PRACTICE 1. Works collaboratively within an interdisciplinary team dedicated to obesity prevention and treatment strategies.

| **Level 1** | **Level 2** | **Level 3** | **Level 4** | **Level 5** |
| --- | --- | --- | --- | --- |
| Limited understanding | Able to describe, in | Able to describe, in | EFFECTIVELY engages | EXEMPLIFIES leadership |
| of the role of the | detail, the scope of | detail, the scope of | multidisciplinary team | within both clinical and |
| physician (both | practice for physicians, | practice for physicians, | members in the clinical | community settings. |
| generalist and | advanced practice | advanced practice | setting to provide | Effectively organizes |
| specialist), advanced | providers, and allied | providers, and allied | comprehensive obesity | medical community |
| practice providers, other | health professionals, but | health professionals, as | treatments, and works | collaboratives to design |
| allied health | INCONSISTENTLY | well as the roles various | collaboratively with | and implement obesity |
| professionals, and | engages | community members, | interdisciplinary team | prevention and |
| community members, | interprofessional team | agencies, and policy | members to advance | intervention initiatives |
| agencies, and policy | members. Has a | makers play in the | obesity prevention and | and guide |
| makers in the prevention | superficial | prevention and | intervention efforts in | multidisciplinary teams |
| and treatment of | understanding of the role | treatment of obesity. | community settings. Has | to impact policy-level |
| obesity. | of various community | Clearly articulates | a SUPERFICIAL | change. |
|  | members, agencies, and | mechanisms in which | understanding of policy- |  |
|  | policy makers in the | interdisciplinary teams | level change processes, |  |
|  | prevention and | work together to achieve | but may begin to |  |
|  | treatment of obesity. | a common goal. Actively | participate in broader |  |
|  |  | participates in | advocacy efforts. |  |
|  |  | multidisciplinary teams |  |  |
|  |  | within the clinical |  |  |
|  |  | setting. |  |  |
|  | | | | |

Unable to evaluate Comment

31* SYSTEMS-BASED PRACTICE 2. Advocates for policies which are respectful and free of weight bias.

| **Level 1** | **Level 2** | **Level 3** | **Level 4** | **Level 5** |
| --- | --- | --- | --- | --- |
| Knowledge of the | Aware of the | Is a role model for peers | Efforts to reduce weight | Effectively utilizes the |
| professional literature | professional literature | in demonstrating | bias within the clinical | professional literature |
| and currently available | and currently available | respectful patient care; | setting are robust; | and currently available |
| resources regarding | resources regarding | proactively seeks to | effectively utilizes the | resources regarding |
| weight bias is limited. | weight bias; however, | reduce weight bias | professional literature | weight bias to advocate |
|  | proactive efforts to | within the clinical | and currently available | on behalf of his/her |
|  | reduce weight bias | setting; however, efforts | resources regarding | patients beyond the |
|  | within the clinical setting | to reduce the effects of | weight bias to educate | clinical setting. This may |
|  | are limited. | weight bias at the | peers; actively engages | include educating |
|  |  | community and policy | other professionals to | community members |
|  |  | levels are limited. | reduce weight bias. | and policy makers or |
|  |  |  |  | lobbying to healthcare |
|  |  |  |  | administrators/ payers |
|  |  |  |  | for resources that |
|  |  |  |  | improve patient |
|  |  |  |  | outcomes and delivery |
|  |  |  |  | of care or decrease |
|  |  |  |  | potential for bias. |
|  | | | | |

Unable to evaluate

Comment

32* SYSTEMS-BASED PRACTICE 3. Utilizes chronic disease treatment and prevention models to advance obesity intervention and prevention efforts within the clinical, community, and public policy domains. Relevant models include social ecological model, social determinants of health, chronic care model, and biopsychosocial model -- clearly articulates the impact of health care delivery systems and accessibility, care coordination, environmental conditions, psychological wellbeing, and various systems of influence (e.g., interpersonal, community, policy) on health and health behaviors.

| **Level 1** | **Level 2** | **Level 3** | **Level 4** | **Level 5** |
| --- | --- | --- | --- | --- |
| Has knowledge of | Able to describe, in | Utilizes populationbased | Effectively and efficiently | Actively advocates for |
| chronic disease | detail, the various | data to drive clinical | coordinates | public policy changes |
| treatment and | chronic disease | practice decision- | comprehensive, patient- | that reduce |
| prevention models is | treatment and | making in the care of | centered care in both | environmental barriers to |
| superficial. | prevention models; | individuals with | clinical and community | health, reduce health |
|  | however, application | overweight or obesity; | settings; application | care systems |
|  | within clinical, | actively engages | within the public policy | inefficiencies, improve |
|  | community, and public | individuals with | domain is limited. | health care accessibility |
|  | policy settings is limited. | overweight or obesity |  | for individuals with |
|  |  | and their families to |  | overweight or obesity, |
|  |  | reduce barriers to health |  | and reduce barriers to |
|  |  | within the environment |  | care coordination |
|  |  | and health care delivery |  | between the health care |
|  |  | systems; however, care |  | team and community |
|  |  | coordination is inefficient |  | agencies. |
|  |  | and limited to health |  |  |
|  |  | care delivery systems; |  |  |
|  |  | application within |  |  |
|  |  | community and public |  |  |
|  |  | policy domains is |  |  |
|  |  | limited. |  |  |
|  | | | | |

Unable to evaluate Comment

33* SYSTEMS-BASED PRACTICE 4. Describes the costs of obesity intervention and prevention with regards to the individual, the health care system, and community.

| **Level 1** | **Level 2** | **Level 3** | **Level 4** | **Level 5** |
| --- | --- | --- | --- | --- |
| Knowledge regarding | Describes, in detail, the | Compares and contrasts | Effectively and efficiently | Has an advanced and |
| the direct, indirect and | direct, indirect, and | the direct, indirect, and | educates peers and | detailed understanding |
| human costs of obesity | human costs of obesity. | human costs of obesity | community members | of the costs of obesity |
| is superficial. | Knowledge regarding | with the costs of obesity | concerning the costs of | and obesity intervention |
|  | the costs of obesity | intervention and | obesity in relation to the | and prevention efforts. |
|  | intervention and | prevention efforts at the | costs of obesity | Participates in cost- |
|  | prevention efforts at the | individual, health care | intervention and | benefit analysis and |
|  | individual, health care | system, community, and | prevention efforts. | contributes to peer- |
|  | system, community, and | population levels. Uses | Applies knowledge of | reviewed literature. |
|  | population levels is | this information to inform | the costs of obesity and | Effectively and efficiently |
|  | limited. | clinical decision-making. | obesity prevention and | educates policy makers |
|  |  |  | intervention to clinical | with regards to the costs |
|  |  |  | decision-making, quality | of obesity in relation to |
|  |  |  | improvement projects, | the costs of obesity |
|  |  |  | and advocacy efforts. | intervention and |
|  |  |  |  | prevention. |
|  | | | | |

Unable to evaluate Comment

BASELINE FELLOW ABILITY IN MANAGING OBESITY-RELATED CO-MORBIDITIES

34* How confident are you in the fellow's performance in Screening and Diagnosis of Obstructive Sleep Apnea?

| Not at all confident. | Not very confident. | Somewhat confident. | Very confident. | Unable to assess. |
| --- | --- | --- | --- | --- |

Comment

35* How confident are you in the fellow's performance in Screening and Diagnosis of ENDOCRINE CONDITIONS including Hypogonadism, Hypothyroidism, and Cushing's Syndrome?

| Not at all confident. | Not very confident. | Somewhat confident. | Very confident. | Unable to assess. |
| --- | --- | --- | --- | --- |

Comment

36* How confident are you in the fellow's performance in performing Screening and Diagnosis of WOMEN'S HEALTH CONDITIONS including Pseudotumor Cerebri, Polycystic Ovary Syndrome, and Menopause?

| Not at all confident. | Not very confident. | Somewhat confident. | Very confident. | Unable to assess. |
| --- | --- | --- | --- | --- |

Comment

37* How confident are you in the fellow's performance in Screening and Diagnosis of Non-Alcoholic Fatty Liver Disease?

| Not at all confident. | Not very confident. | Somewhat confident. | Very confident. | Unable to assess. |
| --- | --- | --- | --- | --- |

Comment

38* How confident are you in the fellow's performance in managing Hypertension?

| Not at all confident. | Not very confident. | Somewhat confident. | Very confident. | Unable to assess. |
| --- | --- | --- | --- | --- |

Comment

39* How confident are you in the fellow's performance in managing Type II Diabetes?

| Not at all confident. | Not very confident. | Somewhat confident. | Very confident. | Unable to assess. |
| --- | --- | --- | --- | --- |

Comment

40* How confident are you in the fellow's performance in managing Dyslipidemia?

| Not at all confident. | Not very confident. | Somewhat confident. | Very confident. | Unable to assess. |
| --- | --- | --- | --- | --- |

Comment

41* How confident are you in the fellow's performance in managing Depression and Anxiety?

| Not at all confident. | Not very confident. | Somewhat confident. | Very confident. | Unable to assess. |
| --- | --- | --- | --- | --- |

Comment

42* How confident are you in the fellow's performance in managing Musculoskeletal Conditions involving joint pain (such as chronic low back pain)?

| Not at all confident. | Not very confident. | Somewhat confident. | Very confident. | Unable to assess. |
| --- | --- | --- | --- | --- |

Comment

43* How confident are you in the fellow's performance in managing medically complex patients? A medically complex patient would be an individual who has multiple chronic conditions, such as type II diabetes, coronary artery disease, history of stroke, etc.

| Not at all confident. | Not very confident. | Somewhat confident. | Very confident. | Unable to assess. |
| --- | --- | --- | --- | --- |

Comment

PERFORMANCE FEEDBACK

44* Describe positive observations of how the fellow performed well over the last month.

45* Do you have any concerns or issues regarding this fellow's performance over the last month? If so, please describe in your comments.

Yes No Comment

46 Describe any suggested areas for improvement that the fellow should address. If possible, please recommend ways to improve.

47* Did you review your evaluation with the fellow?

Yes No Comment

Overall Comment

**Note:** *The evaluator is required to respond to the question.

**Program Evaluation by the Fellow**

**Fellow Assessment of the Obesity Medicine Fellowship Program**

1* How many hours do you typically spend per week at the Clinical Program at Healthful Eating, Activity & Weight Program?
[ ] Range from 0 to 80
Comment

2* In any 2-week period, did you work more than 80 hours per week with less than 1 day off? (If yes, please explain circumstances in comments section).
o Yes
o No
Comments

3* How many vacation days did you take during the last 3 months?
[ ] Range from 0 to 14

4* Overall, how satisfied are you with your clinical experience at the Obesity Medicine Fellowship Program over the last 3 months?
o Very satisfied
o Somewhat satisfied
o Somewhat dissatisfied
o Very dissatisfied
Comment

5* Please tell me how strongly you agree with the following statement... By training at the Obesity Medicine Fellowship Program over the last 3 months, I was helped in achieving the OMEC competencies.
o Strongly agree
o Agree
o Disagree
o Strongly disagree
Comment

6* Please tell me how strongly you agree with the following statement... Over the last 3 months, training at the Obesity Medicine Fellowship Program has helped prepare me for the practice of obesity medicine.
o Strongly agree
o Agree
o Disagree
o Strongly disagree
Comment

7. Please provide any comments or feedback about the Obesity Medicine Fellowship Program.

8*. Please indicate which of the following obesity medicine faculty you have worked with at the Obesity Medicine Fellowship Program over the last 3 months (check all that apply).
o Kimberly Gudzune
o Selvi Rajagopal
o Zoobia Chaudhry
o Craig Hales
o Marci Laudenslager
o Jessica Schwartz
o Larry Cheskin
Other (please specify in comments)
Comment

9* Do you feel that you have had adequate supervision by obesity medicine faculty during the last 3 months at the Obesity Medicine Fellowship Program?
o Yes
o No
Comments

10* Overall, the quality of supervision by the obesity medicine faculty at the Obesity Medicine Fellowship Program over the last 3 months was:
o Excellent
o Good
o Fair
o Poor
Comment

11*. Of the obesity medicine faculty that you have worked with at the Obesity Medicine Fellowship Program over the last 3 months, please indicate the faculty who have POSITIVELY impacted you or your training (check all that apply).
o Kimberly Gudzune
o Selvi Rajagopal
o Zoobia Chaudhry
o Craig Hales
o Marci Laudenslager
o Jessica Schwartz
o Larry Cheskin
o None of the above
o Other (please specify in comments)
Comment

12*. Of the obesity medicine faculty that you have worked with at the Obesity Medicine Fellowship Program over the last 3 months, please indicate any faculty who have NEGATIVELY impacted you or your training (check all that apply).
o Kimberly Gudzune
o Selvi Rajagopal
o Zoobia Chaudhry
o Craig Hales
o Marci Laudenslager
o Jessica Schwartz
o Larry Cheskin
o None of the above
o Other (please specify in comments)
Comment

13. Please provide any comments or feedback about the obesity medicine faculty at the Obesity Medicine Fellowship Program.

Overall Comment

**Faculty Evaluation of the Program**

**FELLOW TRAINING**
For each category below, please indicate your level of agreement.

1. The knowledge and skills gained in the fellowship prepare fellows well for clinical practice as an obesity medicine physician.
☐ Strongly Agree ☐ Agree ☐ Neutral ☐ Disagree ☐ Strongly Disagree ☐ Unable to Assess

2. Program fellows demonstrate a commitment to excellence in their day-to-day activities.
☐ Strongly Agree ☐ Agree ☐ Neutral ☐ Disagree ☐ Strongly Disagree ☐ Unable to Assess

**PROGRAM SUPPORT**
For each category below, please indicate your level of agreement.

3. The program provides adequate communications regarding program goals and fellowship training requirements.
☐ Strongly Agree ☐ Agree ☐ Neutral ☐ Disagree ☐ Strongly Disagree ☐ Unable to Assess

**PROGRAM LEADERSHIP**
For each category below, please indicate your level of agreement.

4. The fellowship program provides adequate opportunities for my own professional development as an obesity medicine physician.
☐ Strongly Agree ☐ Agree ☐ Neutral ☐ Disagree ☐ Strongly Disagree ☐ Unable to Assess
Comments: ______________________________

5. The Program Director provides effective leadership.
☐ Strongly Agree ☐ Agree ☐ Neutral ☐ Disagree ☐ Strongly Disagree ☐ Unable to Assess
Comments: ______________________________

YOUR ROLE AS A FACULTY MEMBER
For each category below, please indicate your level of agreement.

6. Program leadership is responsive to faculty questions and concerns.
☐ Strongly Agree ☐ Agree ☐ Neutral ☐ Disagree ☐ Strongly Disagree ☐ Unable to Assess
Comments: ______________________________

7. I have adequate time for fellow teaching.
☐ Strongly Agree ☐ Agree ☐ Neutral ☐ Disagree ☐ Strongly Disagree ☐ Unable to Assess
Comments: ______________________________

8. I am satisfied with my role in precepting program fellows.
☐ Strongly Agree ☐ Agree ☐ Neutral ☐ Disagree ☐ Strongly Disagree ☐ Unable to Assess
Comments: ______________________________

**ACGME-EQUIVALENT PROGRAM REQUIREMENTS**
For each question below, please indicate either Yes or No.

9. I would recommend participation as faculty in the fellowship program to a professional colleague.
☐ Yes ☐ No
Comments: ______________________________

10. I have received electronic copies on an annual basis of all required GME documents, including the following:
a) The Fellowship Manual which contains GMEC Policies and Procedures for Graduate Medical Education including policies on fellow duty hours, working environment, and moonlighting.
☐ Yes ☐ No
b) Minutes from the semiannual Program Evaluation Committee (PEC) meeting.
☐ Yes ☐ No

11. The evaluation forms that use the OMEC competencies are satisfactory for assessing fellow progress.
☐ Yes ☐ No

12. Please tell us what you feel are the major strengths of the program:
________________________________________________________________________

13. What areas of the program would you suggest for improvement?
________________________________________________________________________

14. Are there ways that the fellowship program could support you as a preceptor?
________________________________________________________________________

**Supplemental Materials 2: Initial and Follow-Up Obesity Medicine Visit Mini-Clinical Evaluation Exercises (Mini-CEXs)**

**Faculty Checklist for Direct Observation of Initial Weight Management Visit conducted by Fellow**

Date of Observation: ________________ Attending: _________________________

Name of Fellow: ____________________________

Year of Training: PGY 4 ___ PGY 5 ___ PGY 6 ___

Estimated # of initial weight management visits conducted by fellow prior to this observation:

___0; ___1-2; ___3-4; ___5-6; ___7-10; ___>10

The fellow has completed a prior faculty-observed initial weight management visit: ___Yes ___ No

Instructions: Observe items below and indicate fellow’s performance with a number from “1-9” as appropriate. See scoring guide on Page 3. If unable to assess a particular item, then indicate in the box labeled “Insufficient Contact to Judge.” Please add comments on Page 2.

| Observation Item # | Skill Being Observed | Unsatisfactory  1—2—3 | Marginal – Needs Attention  4 | Satisfactory  5—6 | Superior  7—8—9 | Insufficient Contact to Judge ( if applicable) |
| --- | --- | --- | --- | --- | --- | --- |
| 1 | Medical History |  |  |  |  |  |
| 2 | Weight History |  |  |  |  |  |
| 3 | Diet History |  |  |  |  |  |
| 4 | Physical Activity History |  |  |  |  |  |
| 5 | Mental Health History |  |  |  |  |  |
| 6 | Sleep History |  |  |  |  |  |
| 7 | Medications History |  |  |  |  |  |
| 8 | Family Weight History |  |  |  |  |  |
| 9 | Surgical History |  |  |  |  |  |
| 10 | Physical Examination |  |  |  |  |  |
| 11 | Humanistic Qualities and Collaborative Skills |  |  |  |  |  |
| 12 | Clinical Judgement and Synthesis When Explaining to Patient |  |  |  |  |  |
| 13 | Documentation Feedback |  |  |  |  |  |
| 14 | Overall Clinical Competence |  |  |  |  |  |

(CEX) Time Spent Observing Fellow: ________Minutes

Total Time Spent Providing Feedback to Fellow: ________Minutes

Lowest Highest

Evaluator’s Level of Satisfaction with Mini-CEX format (from lowest to highest):

1 2 3 4 5 6 7 8 9

Fellow’s Level of Satisfaction with Mini-CEX format(from lowest to highest):

1 2 3 4 5 6 7 8 9

Comment of observing faculty:

Signature of Attending: ____________________________________________ Date:________________________________

Signature of Fellow: ____________________________________________

Date:________________________________

**Faculty Checklist for Direct Observation of Initial Weight Management Visit conducted by Fellow**

**Instructions:** This form and format are for use by the teaching faculty in conducting focused clinical evaluation exercises (CEXs) and/or feedback on Documentation in the fellow continuity practice.

**1. CLINICAL SKILLS – DATA GATHERING (Write in numeric score from 1-9)**

Demonstrates consideration for the patient during the interview. Sets agenda with patient. Recognizes and pursues verbal and nonverbal clues. Allows the patient to tell history without interrupting, yet directs questions smoothly and effectively to obtain pertinent and necessary information. Develops an accurate description of the pertinent symptoms and events. Obtains valid information about medicine-taking. Avoids leading questions, checks on or voices awareness of patient’s milieu (family, events). Determines patient’s ideas/concerns related to weight.

**2. CLINICAL SKILLS – PHYSICAL EXAMINATION (Write in numeric score from 1-9)**

Demonstrates concern for the patient’s comfort and modesty. Enlists the patient’s cooperation. Follows a selective examination appropriate to the patient’s history and specific for weight management visit. Explains what one is examining.

**3. HUMANISTIC QUALITIES AND COLLABORATIVE SKILLS (Write in numeric score from 1-9)**

Demonstrates the necessary qualities and interpersonal skills which foster the development of a therapeutic patient-physician relationship, including personal integrity, compassion, and empathy. Learns about and addresses: patient’s feelings, wishes, concerns, baseline understanding and ideas about symptoms/problem(s) and need for information. Describes options and involves the patient in decision-making. Checks for comprehension and consensus. Assures confidentiality of information disclosed by patient. Includes accompanying person in way(s) preferred by patient. Establishes trust. Places the patient’s problems in the context of the patient’s life and history. Displays sensitivity to the patient’s needs for comfort and encouragement.

**4. CLINICAL JUDGEMENT AND SYNTHESIS WHEN EXPLAINING THINKING TO PATIENT (Write in numeric score from 1-9)**

Spends appropriate time for the complexity of the problem. Keeps focus on one problem at a time. Uses terminology that is meaningful and unambiguous. Presents information concisely, accurately, and in adequate details without significant omissions or digressions. In formulating thinking about nature of problem(s) and about recommended next steps, integrates medical facts, clinical data, and unique facts about patient; weighs alternatives; understand limitations of knowledge. Incorporates consideration of costs, risks, and benefits.

**5. DOCUMENTATION FEEDBACK (Write in numeric score from 1-9)**

- All important subjective and objective findings observed are documented.
- A/P denotes for all active problems the core thinking and plans, including appropriate goal for weight management.
- Medication List is reviewed.
- Patient instructions are written and review key elements of plan.

**6. OVERALL CLINICAL COMPETENCE (as demonstrated in this focused exercise; write in numeric score from 1-9)**

**Faculty Checklist for Direct Observation of Follow-up Weight Management Visit conducted by Fellow**

**Date of Observation: ________________ Attending: __________________________________**

**Name of Fellow: _________________________________**

**Month of Fellowship Training: ______ 1-3 ______ 4-6 ______ 7-12**

**Instructions:** This form and format are for use by the teaching faculty in conducting focused clinical evaluation exercises (CEXs) and/or feedback on documentation in the fellow continuity practice.

**Observe items below and indicate fellow’s performance with a number from “1-9” as appropriate. If unable to assess a particular item, then indicate in the box labeled “Insufficient Contact to Judge.” Please add comments on Page 4.**

**1. CLINICAL SKILLS – DATA GATHERING (Write in numeric score from 1-9)**

Demonstrates consideration for the patient during the interview. Sets agenda with patient. Recognizes and pursues verbal and nonverbal clues. Allows the patient to tell history without interrupting, yet directs questions smoothly and effectively to obtain pertinent and necessary information. Develops an accurate description of the pertinent symptoms and events. Obtains valid information about medicine-taking. Avoids leading questions, checks on or voices awareness of patient’s milieu (family, events). Determines patient’s ideas/concerns related to weight. Reviews patient’s progress with lifestyle interventions, medication changes, and weight trend.

| **Unsatisfactory**  **1—2—3** | **Marginal – Needs Attention**  **4** | **Satisfactory**  **5—6** | **Superior**  **7—8—9** | **Insufficient Contact to Judge**  **( if applicable)** |
| --- | --- | --- | --- | --- |

| **Observed Items** | **Score (1-9)** | **Comments** |
| --- | --- | --- |
| Interim History |  |  |
| Weight Trend |  |  |
| Diet Review |  |  |
| Physical Activity Review |  |  |
| Mental Health Review |  |  |
| Sleep Review |  |  |
| Medications Review |  |  |

**2. CLINICAL SKILLS – PHYSICAL EXAMINATION (Write in numeric score from 1-9)**

Demonstrates concern for the patient’s comfort and modesty. Enlists the patient’s cooperation. Follows a selective examination appropriate to the patient’s history and specific for weight management visit. Explains what one is examining.

| **Unsatisfactory**  **1—2—3** | **Marginal – Needs Attention**  **4** | **Satisfactory**  **5—6** | **Superior**  **7—8—9** | **Insufficient Contact to Judge**  **( if applicable)** |
| --- | --- | --- | --- | --- |

| **Observed Items** | **Score (1-9)** | **Comments** |
| --- | --- | --- |
| Examination as appropriate to patient’s interim  history and review of systems |  |  |

**3. HUMANISTIC QUALITIES AND COLLABORATIVE SKILLS (Write in numeric score from 1-9)**

Demonstrates the necessary qualities and interpersonal skills which foster the development of a therapeutic patient-physician relationship, including personal integrity, compassion, and empathy. Learns about and addresses: patient’s feelings, wishes, concerns, baseline understanding and ideas about symptoms/problem(s) and need for information. Describes options and involves the patient in decision-making. Checks for comprehension and consensus. Assures confidentiality of information disclosed by patient. Includes accompanying person in way(s) preferred by patient. Establishes trust. Places the patient’s problems in the context of the patient’s life and history. Displays sensitivity to the patient’s needs for comfort and encouragement.

| **Unsatisfactory**  **1—2—3** | **Marginal – Needs Attention**  **4** | **Satisfactory**  **5—6** | **Superior**  **7—8—9** | **Insufficient Contact to Judge**  **( if applicable)** |
| --- | --- | --- | --- | --- |

Score: _____ (1-9)

**4. CLINICAL JUDGEMENT AND SYNTHESIS WHEN EXPLAINING THINKING TO PATIENT (Write in numeric score from 1-9)**

Spends appropriate time for the complexity of the problem. Keeps focus on one problem at a time. Uses terminology that is meaningful and unambiguous. Presents information concisely, accurately, and in adequate details without significant omissions or digressions. In formulating thinking about nature of problem(s) and about recommended next steps, integrates medical facts, clinical data, and unique facts about patient; weighs alternatives; understand limitations of knowledge. Incorporates consideration of costs, risks, and benefits.

| **Unsatisfactory**  **1—2—3** | **Marginal – Needs Attention**  **4** | **Satisfactory**  **5—6** | **Superior**  **7—8—9** | **Insufficient Contact to Judge**  **( if applicable)** |
| --- | --- | --- | --- | --- |

| **Observed Items from Management Plan** | **Score (1-9)** | **Comments** |
| --- | --- | --- |
| Assessment of weight progress |  |  |
| Specific and time-oriented weight loss goal  incorporating patient’s input and  personal and/or clinical health goals |  |  |
| Nutrition |  |  |
| Physical Activity |  |  |
| Stress/Mental Health |  |  |
| Sleep |  |  |
| Review and plan for weight-promoting  medications |  |  |
| Anti-obesity medications |  |  |
| Bariatric Procedures |  |  |
| Co-morbidities assessment/plan |  |  |
| Diagnostic Studies |  |  |

**5. DOCUMENTATION FEEDBACK (Mark as Satisfactory/Unsatisfactory)**

(For use with EPIC EMR):

| **Items** | **Feedback** |
| --- | --- |
| All important subjective and objective findings  observed are documented. | ____  _______ Satisfactory _______Unsatisfactory |
| A/P denotes for all active problems the core  thinking and plans, including appropriate goal for weight management. | ____  ____. _______ Satisfactory _______Unsatisfactory |
| Medication List is reviewed. | ____  __. _______ Satisfactory _______Unsatisfactory |
| Patient instructions are written and review key  elements of plan. | ____  ------- _______ Satisfactory _______Unsatisfactory |

**6. BILLING** **(Mark as Satisfactory/Unsatisfactory)**

_______ Satisfactory _______Unsatisfactory

**7. OVERALL CLINICAL COMPETENCE (as demonstrated in this focused exercise; write in numeric score from 1-9)**

| **Unsatisfactory**  **1—2—3** | **Marginal – Needs Attention**  **4** | **Satisfactory**  **5—6** | **Superior**  **7—8—9** | **Insufficient Contact to Judge**  **( if applicable)** |
| --- | --- | --- | --- | --- |

Score: _____ (1-9)

(CEX) Time Spent Observing Fellow: ________Minutes

Total Time Spent Providing Feedback to Fellow: ________Minutes

Evaluator’s Level of Satisfaction with Mini-CEX format (from lowest to highest):

1 2 3 4 5 6 7 8 9

Fellow’s Level of Satisfaction with Mini-CEX format(from lowest to highest):

1 2 3 4 5 6 7 8 9

**Comment of observing faculty:**
